# Differential Effects of APOE Genotype on MicroRNA Cargo of Cerebrospinal Fluid Extracellular Vesicles in Females With Alzheimer’s Disease Compared to Males

**DOI:** 10.3389/fcell.2022.864022

**Published:** 2022-04-27

**Authors:** Ursula S. Sandau, Trevor J. McFarland, Sierra J. Smith, Douglas R. Galasko, Joseph F. Quinn, Julie A. Saugstad

**Affiliations:** ^1^ Department of Anesthesiology and Perioperative Medicine, Oregon Health and Science University, Portland, OR, United States; ^2^ Department of Neurosciences, University of California, San Diego, La Jolla, CA, United States; ^3^ Department of Neurology, Oregon Health and Science University, Portland, OR, United States; ^4^ Parkinson Center and Movement Disorders Program, Oregon Health and Science University, Portland, OR, United States; ^5^ Portland VAMC Parkinson’s Disease Research, Education, and Clinical Center, Portland, OR, United States

**Keywords:** Alzheimer’s disease, cerebrospinal fluid (CSF), extracellular vesicle (EV), microRNA, apolipoprotein E allele (APOE), sex difference

## Abstract

Multiple biological factors, including age, sex, and genetics, influence Alzheimer’s disease (AD) risk. Of the 6.2 million Americans living with Alzheimer’s dementia in 2021, 3.8 million are women and 2.4 million are men. The strongest genetic risk factor for sporadic AD is apolipoprotein E-e4 (APOE-e4). Female APOE-e4 carriers develop AD more frequently than age-matched males and have more brain atrophy and memory loss. Consequently, biomarkers that are sensitive to biological risk factors may improve AD diagnostics and may provide insight into underlying mechanistic changes that could drive disease progression. Here, we have assessed the effects of sex and APOE-e4 on the miRNA cargo of cerebrospinal fluid (CSF) extracellular vesicles (EVs) in AD. We used ultrafiltration (UF) combined with size exclusion chromatography (SEC) to enrich CSF EVs (e.g., Flotillin+). CSF EVs were isolated from female and male AD or controls (CTLs) that were either APOE-e3,4 or -e3,3 positive (n = 7/group, 56 total). MiRNA expression levels were quantified using a custom TaqMan™ array that assayed 190 miRNAs previously found in CSF, including 25 miRNAs that we previously validated as candidate AD biomarkers. We identified changes in the EV miRNA cargo that were affected by both AD and sex. In total, four miRNAs (miR-16-5p, -331-3p, -409-3p, and -454-3p) were significantly increased in AD vs. CTL, independent of sex and APOE-e4 status. Pathway analysis of the predicted gene targets of these four miRNAs with identified pathways was highly relevant to neurodegeneration (e.g., senescence and autophagy). There were also three miRNAs (miR-146b-5p, -150-5p, and -342-3p) that were significantly increased in females vs. males, independent of disease state and APOE-e4 status. We then performed a statistical analysis to assess the effect of APOE genotype in AD within each sex and found that APOE-e4 status affects different subsets of CSF EV miRNAs in females vs. males. Together, this study demonstrates the complexity of the biological factors associated with AD risk and the impact on EV miRNAs, which may contribute to AD pathophysiology.

## Introduction

Alzheimer’s disease (AD) is the most common form of dementia, the sixth leading cause of death in the United States, and the fifth leading cause of death for those age 65 and older (Alzheimer’s Association, 2021). In 2021, an estimated 6.2 million Americans age 65 and older are living with Alzheimer’s dementia, a number that is projected to reach 13.8 million by 2060 (Alzheimer’s Association, 2021). The costs of health- and long-term care for individuals with Alzheimer’s dementias are substantial: total payments in 2021 for all individuals with Alzheimer’s or other dementias are estimated at $355 billion, not including value for informal caregiving, and these costs will increase by $1 billion each year (Alzheimer’s Association, 2021). As AD is a global disease, these projections make dementia one of the costliest conditions to society (Alzheimer’s Association, 2021).

There are multiple biological factors, including age, sex, and genetics, that influence AD risk (Alzheimer’s Association, 2021). Of the 6.2 million Americans living with Alzheimer’s dementia, 3.8 million are women and 2.4 million are men (Rajan et al., 2021). Thus, more women than men have Alzheimer’s or other dementias, and almost two-thirds of Americans with AD are women (Rajan et al., 2021). The most important genetic risk factor for sporadic AD is apolipoprotein E (APOE), a major lipoprotein in the central nervous system (CNS) that is associated with triglyceride-rich lipoproteins and mediates the clearance of these lipoproteins from the plasma (Corder et al., 1993; Levy and Levy, 2021). Of the three major APOE gene alleles (e2, e3, and e4), the APOE-e4 allele is the strongest risk factor for AD. Importantly, while the APOE-e2 allele is relatively rare and may provide some protection against AD (Reiman et al., 2020), people who inherit one copy of the APOE-e4 allele have an increased chance of developing the disease; those who inherit two copies of the allele are at even greater risk (Sando et al., 2008; Reiman et al., 2020). In addition, female APOE-e4 carriers are more likely to progress from mild cognitive impairment (MCI) to AD, have more brain atrophy and memory loss, and develop AD more frequently than age-matched males (Fleisher et al., 2008; Altmann et al., 2014; Sampedro et al., 2015). Consequently, biomarkers that are sensitive to biological risk factors may improve diagnostics and provide insight to underlying mechanistic changes that could drive AD progression.

In our prior studies, we focused on the utility of extracellular miRNAs as biomarkers for AD in total CSF that was not fractionated to separate and enrich for EVs. We initially discovered a set of 36 miRNAs in CSF from living donors that could classify AD patients from healthy controls (CTLs) (Lusardi et al., 2017). Our validation study in CSF from a new and independent cohort of AD patients and CTLs showed that 25 of the 36 biomarker candidates serve as classifiers for AD (Wiedrick et al., 2019). We then assessed whether any of the validated CSF miRNAs are sensitive to early-stage pathology as exemplified by MCI diagnosis. We observed that five miRNAs showed a linear trend of decreasing median expression across the ordered diagnoses (CTL to MCI to AD) (Sandau et al., 2020b). Importantly, three of these five trending miRNAs (miR-142-3p, -146a-5p, and -146b-5p) have been identified as candidate biomarkers for MCI and/or AD in total CSF in other studies (Cogswell et al., 2008; Alexandrov et al., 2012; Kiko et al., 2014; Denk et al., 2015; Nagaraj et al., 2017; Park et al., 2019).

Extracellular miRNAs in CSF have multiple carrier types, including extracellular vesicles (EVs), RNA-binding proteins, and high-density lipoproteins (Vickers and Remaley, 2012; Mori et al., 2019). EVs are membrane-bound spheres that carry complex cargos, including lipids, proteins, and nucleic acids (Colombo et al., 2013). The release of EVs is a universally conserved cellular process that occurs in all eukaryotes and prokaryotes and in every biofluid examined. EVs released from CNS cells contribute to cell-to-cell communication throughout the CNS and the periphery (Chivet et al., 2012; Dickens et al., 2017; Zhang and Yang, 2018) in normal and pathological processes (Yuyama and Igarashi, 2016; Neven et al., 2017). Thus, there is interest in exploring the molecular cargo of EVs in biofluids as biomarkers for AD that may 1) aid in tracking disease progression including diagnosis during the prodromal stage, 2) differentiate AD patients from other neurodegenerative disorders that also secrete EVs, and 3) identify new therapeutic targets (Rastogi et al., 2021).

Cells produce three main types of EVs classified by their size and mode of release from cells (Raposo and Stoorvogel, 2013; Kim et al., 2017). Apoptotic bodies are ∼500–2000 nm in diameter and are released *via* blebbing of the plasma membrane, while microvesicles (MVs) are ∼150–1,000 nm and are released *via* budding of the plasma membrane. In contrast, exosomes measure ∼40–150 nm and arise from the endosomal pathway, which forms intracellular multivesicular bodies (MVB) that are secreted as vesicles into the extracellular space. Two additional nanoparticle types, exomeres and supermeres, have also been recently identified in cultured cells (Zhang et al., 2018; Zhang et al., 2021). Importantly, to date all of the EV subtypes and small nanoparticles have distinct RNA profiles (Crescitelli et al., 2013; Mori et al., 2019; Zhang et al., 2021). Furthermore, EVs have protein cargo and surface marker profiles that are indicative of their respective biogenesis routes and cellular origin (e.g., neuron vs. astrocyte). Importantly, in AD, disruptions in the endolysosomal pathway affect exosome biogenesis, including the RNA and protein cargo, which can consequently be exploited as biomarkers (Mathews and Levy, 2019). While only four studies have profiled CSF EV miRNAs by either RNA sequencing or comprehensive qPCR arrays (Gui et al., 2015; Riancho et al., 2017; McKeever et al., 2018; Jain et al., 2019), the expression levels for two (miR-16-5p and -125b-5p) of our 25 validated biomarkers were altered in CSF EVs of AD participants, relative to CTLs (Gui et al., 2015; McKeever et al., 2018; Wiedrick et al., 2019). There is also evidence that APOE-e4 mediates disruptions in endolysosomal pathways and reduces brain exosome levels in aged, non-AD human brain (Peng et al., 2019). However, broad profiles of the types of EV and their cargos are yet to be defined in cerebrospinal fluid (CSF) from AD participants while also taking into account the APOE-e4 status and sex.

Here, we sought to establish the effect of AD on the miRNA cargo of CSF EVs and determine whether AD risk factors (sex and APOE genotype) also impact EV miRNA expression. We first established a protocol using living donor’s CSF and a combined approach of ultrafiltration (UF) plus size exclusion chromatography (SEC) to enrich EVs positive for canonical markers, such as Flotillin, TSG101, and CD81, while also depleting a majority of proteins and lipoproteins not associated with EVs. We then used UF plus SEC to isolate EVs from females and males that were either AD or CTLs and were either APOE-e3,4 or APOE-e3,3 positive. We quantified miRNA expression levels in the isolated EVs using a custom TaqMan**™** qPCR array with probes for 190 miRNAs previously found in CSF, including the 25 miRNAs we validated as candidate biomarkers for AD (Wang et al., 2017; Wiedrick et al., 2019). We identified EV miRNAs whose expression levels were affected by both AD and sex of the participants. Furthermore, we found that APOE-e4 status affects different subsets of CSF EV miRNAs in females vs. males. Thus, the miRNA cargo of CSF EVs is informative for neurological disorders and sensitive to both sex and genotype. Together, these studies demonstrate the complexity of the biological factors associated with AD risk, and their impact the EV cargo, which may play a mechanistic role in AD pathophysiology.

## Materials and Methods

### Study Participants

**FIGURE 1:**
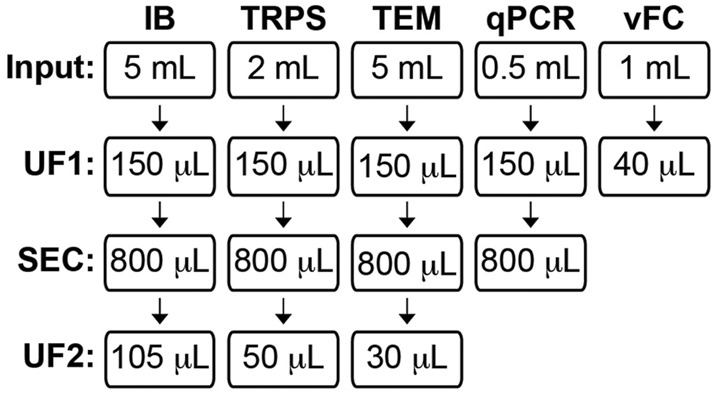
Human CSF EV study workflow. Human CSF was assessed using five independent methods: immunoblot (IB), tunable resistive pulse sensing (TRPS), transmission electron microscopy (TEM), qPCR for miRNA analysis, and vesicle flow cytometry (vFC). The CSF volume input and subsequent processing step(s) prior to assaying are shown for each technique. CSF for IB, TRPS, and TEM was processed by ultrafiltration (UF1), followed by size exclusion chromatography (SEC) to collect an 800 µL pool of fractions (Fxs) 6–9, and post-SEC UF (UF2) to concentrate the pool. CSF for miRNA qPCR was processed by UF1 and then SEC, and RNA was isolated from an 800 µL pool of Fxs 6–9. CSF for vFC was processed by UF-only to concentrate the biofluid prior to being assayed.

For experiments that characterized CSF EVs by immunoblot, tunable resistive pulse sensing (TRPS), transmission electron microscopy (TEM), and single vesicle flow cytometry (vFC) (Figure 1), we pooled human CSF from neurologically normal male and female participants obtained from the Oregon Health & Science University (OHSU) Oregon Alzheimer’s Disease Research Center (OADC), core program of the OHSU Layton Aging & Alzheimer’s Disease Center or purchased from BioChemed (Winchester, VA). For CSF EV miRNA quantification by qPCR, we used individual human CSF samples from 28 CTL and 28 AD participants (Figure 1; Table 1) obtained from the OADC or from the University of California San Diego Shiley-Marcos Alzheimer’s Disease Research Center (UCSD ADRC). All procedures were approved by the Institutional Review Boards of OHSU (IRB 6845) and UCSD (IRB 80012). All participants at both the institutions provided written informed consent and underwent detailed evaluations consisting of medical history, physical and neurological examinations, laboratory tests, and neuropsychological assessments, including cognitive tests and interview with a collateral historian. CTL participants were volunteers in good health with no symptoms of cognitive impairment or neurological disease and had normal performance on a detailed battery of neuropsychological tests. Sex, age, MMSE, and APOE genotype (e3,3 or e3,4) status for all 56 participants in this study are shown in Table 1.

**TABLE 1:** CSF participant characteristics. The table includes sex, age, and MMSE measures of the 56 participants in this study. The ratios of APOE genotype (-e3,3 or e-3,4) and sex was matched across all groups.

	CTL e-3,3	CTL e-3,4	AD e-3,3	AD e-3,4
SEX				
Female	7 (50%)	7 (50%)	7 (50%)	7 (50%)
Male	7 (50%)	7 (50%)	7 (50%)	7 (50%)
Total	14	14	14	14
AGE (years)^a^	Mean ± SD			
Female	71.0 ± 5.8	70.9 ± 3.7	73.4 ± 12.4	73.3 ± 5.1
Male	72.6 ± 8.5	73.3 ± 9.5	72.4 ± 7.9	73.1 ± 4.5
Total	71.8 ± 7.0	72.1 ± 7.0	72.9 ± 10.0	73.2 ± 4.6
MMSE^a^	Mean ± SD			
Female	29.6 ± 0.8	29.6 ± 0.8	21.9 ± 4.0	21.3 ± 2.5
Male	29.4 ± 0.8	28.6 ± 2.2	21.8 ± 3.6	21.1 ± 2.7
Total	29.5 ± 0.8	29.1 ± 1.7	21.8 ± 3.6	21.2 ± 2.2
				

a Age and MMSE values represent data at the time of CSF collection.

### CSF Collection

The OADC and USCD ADRC both used a standardized CSF collection protocol corresponding to that of other AD centers engaged in biomarker research (Shi et al., 2011). Lumbar punctures were done in the morning under fasting conditions in the lateral decubitus position with a 24-gauge Sprotte spinal needle. The OADC sends the first 3–5 ml of CSF collected to the clinical lab for cell counts and determination of glucose and total protein levels, and the UCSD ARDC sends the first 2 ml of CSF collected to the clinical lab for testing. At both sites, serial syringes with 5 ml of CSF were collected, mixed, and transferred to polypropylene tubes in 0.5-ml aliquots, and the tubes were numbered to account for any gradient effect in subsequent experiments. The tubes were labeled with a subject number, but no other identifying information was mentioned. The CSF aliquots were flash frozen on dry ice and stored at −80°C. All CSF samples were only thawed once on ice prior to use.

### APOE-e4 Genotyping

APOE-e4 genotypes at OHSU were determined by sequencing the amplicon of APOE exon 4 (e4 allele). Genomic DNA was isolated from blood and amplified by Touchdown PCR with 250 µM dNTPs, 1 unit Taq DNA polymerase, buffer, 1X Q-solution (Qiagen, Valencia, CA), and 0.5 µM forward (5′-GGC​GCT​GAT​GGA​CGA​GAC​C-3′) and reverse (5′-CCT​GGG​CCC​GCT​CCT​GTA​G-3′) primers to amplify the APOE exon 4. A product size of 443 nucleotides identified on a 1% agarose 1X TBE gel was excised, cleaned with ExoSAP-IT reagent (Affymetrix), and sequenced on a model 377 automated fluorescence sequencer (Applied Biosystems). Chromatogram traces were examined, and nucleotide sequences were determined using FinchTV (Geospiza, Inc.). APOE-e4 genotypes at UCSD were determined by PCR amplification and restriction fragment length polymorphism (RFLP) (Hixson and Vernier, 1990), as previously described (Wierenga et al., 2012). Venous blood was drawn from participants and genomic DNA was extracted using the QIAamp DNA Blood Mini Kit (Qiagen) followed by PCR amplification. The APOE-e4 gene sequences were amplified using forward (5′-ACG​CGG​GCA​CGG​CTG​TCC​AAG​GA-3′) and reverse (5′-GCG​GGC​CCC​GGC​CTG​GTA​CAC-3′) primers. The amplification products were digested with *HhaI* (restriction enzyme site GCG^C) and then subjected to electrophoresis on polyacrylamide gels; the gels were stained with ethidium bromide, and the digested fragments were visualized by ultraviolet illumination. A unique combination of *HhaI* fragment sizes enabled typing of all homozygotic and heterozygotic combinations: *HhaI* cleaves at GCGC encoding 112arg (e4) and 158arg (e3, e4) but does not cut at GTGC-encoding 112cys (e2, e3) and 158cys (e2).

### Single vFC of CSF

For single vFC studies, we examined total CSF that was not concentrated as well as 1.0 ml of CSF that was concentrated by UF using Microcon®-100 kDa Centrifugal Filters (MPE100025, Millipore Sigma, Burlington, MA) to a final volume of 40 μL (25x). We analyzed the CSF by single vFC using fluorescence to estimate vesicle size, concentration, and surface cargo with a commercial flow cytometer (Beckman Coulter CytoFlex S, Brea, CA) and an assay kit (vFC™ EV Analysis kit, Cellarcus Biosciences, San Diego, CA). In brief, 5 µL each of the unconcentrated and 25x concentrated CSF were stained according to the manufactures recommendations with a membrane stain (vFRed) and pool of fluorescence-labeled antibodies against tetraspanins (TS PE mix: CD9, CD63, and CD81) for 1 h at room temperature. The stained samples were diluted 1:1,000 prior to reading on the flow cytometer and detected using fluorescence triggering (excitation: 488 nm; emission: 690/50 nm). To demonstrate the vesicular nature of the vFRed and/or TS-positive (TS+) events, an aliquot of the stained, concentrated 25x CSF was detergent treated (0.05% Triton X-100) for 1 min prior to being assayed. Data analysis was performed using FCS Express (Version 7, De Novo Software, Pasadena, CA). Events were gated with respect to time (to eliminate spurious background that occurs at the start of each sample), vFRed pulse shape (to eliminate short pulse width background events), and violet side scatter (VSSC) vs. vFRed fluorescence (to include events with characteristic membrane fluorescence and light scatter) (Supplementary Figure S4). NanoRainbow beads (Cellarcus Biosciences) were used to characterize critical performance metrics that enable the evaluation of laser alignment and fluorescence resolution (Supplementary Figure S1). Membrane fluorescence was calibrated in terms of vesicle size (surface area) using a synthetic vesicle size standard (Lipo100 beads, Cellarcus Biosciences), and diameter was calculated assuming a spherical shape, resulting in an acceptable size distribution, in accordance to Cellarcus Biosciences (Supplementary Figure S3A). Standardized preparations of platelet EVs were used as reference samples for surface marker immunofluorescence (Supplementary Figure S3B). Particle concentrations were determined by subtracting the background values of buffer control containing vFRed and TS PE Mix (without CSF) from the experimental sample values.

### CSF EV Isolation by UF and SEC

EVs were isolated from CSF by a combined approach of UF and SEC for downstream use in immunoblots, TRPS, TEM, and miRNA qPCR arrays according to Figure 1. Depending on the downstream application, aliquots of 0.5, 2.0, or 5.0 ml of CSF were thawed on ice, pooled, and then concentrated by UF using a 0.5-ml Microcon®-30 kDa Centrifugal Filters (MRCF0R030, Millipore Sigma) and centrifugation at 14,000 x g for 15 min at 4°C. Concentrated samples were recovered by inverting the centrifugal filter into a clean collection tube and then centrifuged at 2,000 x g for 3 min at 4°C. The concentrated CSF was then brought to a final volume of 150 µL with 0.22-µm-filtered PBS. CSF EVs were isolated from the concentrated CSF by SEC using qEV single 35 nm or 70 nm columns (IZON Science, Christchurch, New Zealand), according to the manufacturer’s recommendations. Prior to use the qEV columns were brought to room temperature and equilibrated with 0.22-µm-filtered PBS. The void volume of the column consisting of fractions (Fxs) 1–5 (1 ml) and pools of subsequent Fxs 6–9, 10–13, and 14–17 (800 μL/pool) were collected. For immunoblot, TRPS, and TEM, each pool was then UF using a 0.5-ml Microcon®-30 kDa Centrifugal Filter to a final volume of 105 μL for immunoblot, 50 μL for TRPS, and 30 μL for TEM, as described before. For qPCR, the 800 μL pools were directly processed for RNA isolation without a second UF. For immunoblot, TRPS, and qPCR, the pools were frozen at −80°C until use. For TEM, the pools were stored on wet ice or 4°C until processing within 6 h. All centrifugation steps in this subsection of the methods were conducted using the Microfuge 22R centrifuge equipped with an F241.5P fixed angle rotor (Beckman Coulter).

### Immunoblot Evaluation of EV Markers in Pools of SEC Fxs

Protein concentrations were measured using the Qubit protein assay kit and Qubit 4 fluorometer (Thermo Fisher Scientific, Waltham, MA) for the concentrated UF-SEC pools using 5 μL of the void and Fxs 6–9, 1 μL of Fxs 10–13, and 1 μL of 1:10 diluted Fxs 14–17. The remaining volume of each pool was prepared for SDS-PAGE by diluting in 4x NuPAGE™ LDS sample buffer (Thermo Fisher Scientific). To assess the separation of EV associated proteins from vesicle-free proteins and lipoproteins, we performed a total protein stain and immunoblots for APOA1, APOE, and albumin by loading equal volumes (37 μL) of the void, Fxs 6–9, 10–13, and 14–17 on a NuPAGE™ 4–12% Bis-Tris 1.5 mm x 10 well gels (Thermo Fisher Scientific). To identify the SEC Fxs that contained an enrichment of EV markers, equal concentrations (0.1 μg) of void, Fxs 6–9, 10–13, 14–17, and neurologically normal human frontal cortex lysate (positive control) were run on 4–12% gels, as described above, and immunoblotted for CD9, CD63, CD81, flotillin, TSG101, annexin V (AnnV), synaptophysin (SYP), NCAM-1, GLAST, CD11b, and TMEM119 proteins. In addition, gels loaded with 1.0 µg of void, Fxs 6–9, 10–13, 14–17, and human frontal cortex were immunoblotted for SYP and CD11b. Following electrophoresis, proteins were transferred to PVDF membranes and kept overnight at 4°C (30 V constant current). Membranes were incubated for 1 h with a blocking buffer (5% non-fat milk in TBS with 0.05% Tween 20 (TBST)) and then incubated with primary antibody diluted with TBST overnight at 4°C, and for 1 h with secondary antibody (1:10,000 TBST) at room temperature. Membranes were developed using the West Dura, Pico, or Femto chemiluminescence kits (Thermo Fisher Scientific) and imaged using the ChemiDoc Touch imaging system (Bio-Rad, Hercules, CA). The following antibodies were used for immunoblotting: albumin 1:1,000 (#4929, Cell Signaling Technology, Danvers, MA), APOA1 (12C8) 1:200 (sc-080551, Santa Cruz Biotechnology, Dallas, TX), APOE 1:2000 (50A-G1A, Academy Bio-medical Company, Inc., Houston, TX), AnnV 1:5,000 (GTX103250, GeneTex, Irvine, CA), CD9 (C-4) 1:200 (sc-13118, Santa Cruz Biotechnology), CD11b 1:1,000 (ab133357, Abcam, Cambridge, United Kingdom), CD63 1:1,000 (ab134045, Abcam), CD81 (B-11) 1:100 (sc-166029, Santa Cruz Biotechnology), flotillin 1:10,000 (ab133497, Abcam), GLAST 1:500 (NB100-1869, Novus Biologicals, Littleton, CO), NCAM-1 1:125 (NBP2-38452, Novus Biologicals), SYP 1:1,000 (#36406, Cell Signaling Technology), TMEM119 (#66948-1-Ig, Proteintech, Rosemont, IL), and TSG101 1:1,000 (ab125011, Abcam). Secondary antibodies were purchased from Jackson ImmunoResearch (West Grove, PA) and included horseradish peroxidase (HRP)–conjugated donkey anti-mouse (#715-035-150), donkey anti-rabbit (#711-035-152), and donkey anti-goat (#705-035-003).

### TRPS of SEC Pools

Pools of the void volume (Fxs 1–5) and Fxs 6–9 were analyzed for particle size and concentration by TRPS using a qNANO gold instrument fitted with a NP80 nanopore (IZON Science). Prior to TRPS, 800 µL SEC pools were subjected to UF using Microcon®-30 kDa centrifugal filters and then brought to a final volume of 50 µL in 2x PBS (Figure 1). TRPS measurements were recorded with the NP80 stretched to 47.50 mm and using 0.34 V at a pressure reading of 5 (mbar). Each sample was analyzed until at least 500 particles were recorded or until the maximum recording time of 10 min elapsed. Data were acquired and analyzed using the IZON Control Suite version 3.4 software (IZON Science). The size (nm) and concentration (particles/mL) of particles measured in the SEC pools were calibrated against CPC100 calibration beads (IZON Science).

### TEM of SEC Pools

Pools of CSF SEC fractions including the void, Fxs 6–9, 10–13, and 14–17 were concentrated by UF to a final volume of 30 μL and then stored on wet ice until processing for TEM within 6 h (Figure 1) by the OHSU Multiscale Microscopy Core. A volume of 5 µL of the SEC preparations were deposited on glow-discharged (120 s 15 mAmp, negative mode) carbon formvar 400 mesh copper grids (01822-F, Ted Pella, Inc., Redding, CA) for 3 min, rinsed 15 s in water, wicked on Whatman filter paper 1, stained for 60 s in filtered 1.33% (w/v) uranyl acetate in water, wicked again, and air dried. Samples were imaged by using the Multiscale Microscopy Core at 120 kV on a FEI Tecnai™ Spirit TEM system (Thermo Fisher Scientific Electron Microscopy, Hillsboro, OR). Images were acquired as 2048 × 2048 pixel, 16-bit gray scale files using the FEI’s TEM Imaging & Analysis interface on an Eagle™ 2K CCD multiscan camera.

### CSF EV RNA Isolation and MiRNA Arrays

EVs were isolated from 500 μL of CSF that was first concentrated by UF to 150 μL then fractionated by SEC (Figure 1). Fxs 6–9, 10–13, and 14–17 were pooled to final volume of 800 μL, and total RNA was isolated using the mirVana™ PARIS™ RNA and a Native Protein Purification Kit (AM1556, Thermo Fisher Scientific), with modification (Burgos et al., 2014), as we have previously reported for CSF and plasma EVs (Lusardi et al., 2017; Wiedrick et al., 2019; Sandau et al., 2020a; Sandau et al., 2020b; Lusardi et al., 2021). Isolated RNA was then stored at −80°C until use. For qPCR, the RNA samples were concentrated (R1013, RNA Clean and Concentrator™-5 Kit, Zymo Research, Irvine, CA) and then eluted into 6 μL of RNase/DNase-free water. Total RNA was assayed on Custom TaqMan™ Advanced Array Cards (192a Format) that include probes for 191 human CSF miRNAs, plus five endogenous non-changing miRNA controls used for normalization: miR-191-5p, -204-3p, -204-5p, -342-3p, and -574-3p (Supplementary Table S1). Using a pool of Custom RT Primers for the CSF array (Thermo Fisher Scientific) and MultiScribe™ Reverse Transcriptase (4, 311, 235, Thermo Fisher Scientific), 3.2 μL of concentrated RNA was reverse transcribed in a total reaction volume of 7.5 μL. A volume of 5 μL of cDNA was pre-amplified (PreAmp) for 14 cycles using Custom PreAmp Primers for the CSF array (Thermo Fisher Scientific) and TaqMan™ PreAmp Master Mix (4, 391, 128, Thermo Fisher Scientific) in a final reaction volume of 25 μL. Reverse transcription and PreAmp reactions were run with a Bio-Rad T100 thermal cycler (Thermo Fisher Scientific), following the manufacturer’s instructions, for detection of miRNAs with PreAmp. All cDNA was stored at −20°C. Prior to qPCR the PreAmp cDNA was diluted to 1:2 in RNase/DNase-free water and 18 μL was mixed with TaqMan™ Universal Master Mix II, no UNG (444,047, Thermo Fisher Scientific). The samples were loaded on the custom CSF arrays, and the qPCR amplifications and data acquisition were carried out on a QuantStudio™ 12K Flex real-time PCR system (Thermo Fisher Scientific).

### CSF EV MiRNA Expression Analysis

MiRNAs were analyzed using relative quantification (∆∆Cq) based on Applied Biosystems recommendations (Thermo Fisher Scientific, Part Number 4371095 Rev B). Cq values were calculated using automatic baseline, and threshold values were determined by ExpressionSuite Software v.1.3 (Thermo Fisher Scientific). The Cq value for each well was reported along with the amplification score (AmpScore) and a Cq confidence (CqConf), which are metrics for the quality of each amplification, as we have previously reported (Lusardi et al., 2017; Wiedrick et al., 2019; Sandau et al., 2020a; Sandau et al., 2020b; Lusardi et al., 2021). Prior to data analysis, amplifications were filtered according to each well’s Cq value, AmpScore, and CqConf: 1) PCR products with a Cq > 34, or reported as “Undetected”, were considered below the detection threshold and assigned a Cq value of “34”; *2*) amplifications with a Cq ≤ 34 and an AmpScore <1.0 or a CqConf <0.8 were excluded from analysis. Based on these criteria, miRNAs with a Cq ≤ 34, AmpScore >1.0, and CqConf >0.8 were deemed considered for further analysis. Next, we excluded miRNAs that were not expressed in at least 20% of the samples (11 out of 56), which would enable analysis of miRNAs differentially detected in one out of the eight subgroups (e.g., APOE-e3,4 females with AD). We also performed qPCR reactions with water only (no RNA—no template control) as a control for spurious PCR amplifications (Supplementary Table S2). MiRNAs that had good quality amplifications (Cq ≤ 34, AmpScore >1.0, and CqConf >0.8) in the water only control that were within 1 Cq of the average Cq for the 56 experimental samples were excluded from downstream analysis. For miRNAs that did meet all of the inclusion criteria, we then used the following formula for calculating the ∆∆Cq for each miRNA: ∆∆Cq = mean ∆Cq of the test samples (e.g., AD) − mean ∆Cq of reference samples (e.g., CTL). Within each sample, the ∆Cq for a miRNA was calculated by the following formula: ∆Cq = miRNA Cq − mean Cq of endogenous control miRNAs. MiRNAs selected as endogenous control normalizers showed 1) stable good quality expression values in all samples regardless of experimental group and 2) best endogenous control scores identified in ExpressionSuite. For each miRNA, the fold change (RQ value) was calculated by the 2^−ΔΔCq^ method, with RQ > 1 indicating increased miRNA expression in either AD vs. CTL or females vs. males. Conversely, RQ < 1 indicates decreased expression in AD or females, compared to their respective reference group. The miRNA microarray data is MIAME compliant and submitted to the Gene Expression Omnibus site: ncbi.nlm.nih.gov/geo/.

### CSF EV MiRNA Statistical Analysis

Data were analyzed with GraphPad Prism software v9.3.1 (GraphPad Software, Inc., San Diego, CA). Data are shown as either a Volcano plot (−Log10 (q-value) vs. the Log2(Fold change)) or the ΔCq mean ± standard error mean (SEM). Where possible the biological replicates are displayed as individual symbols. To assess the effects of AD on miRNA levels, participants were categorized as either AD (n = 28) or CTL (n = 28) and analyzed by Welch’s unpaired *t*-test with a Benjamini–Hochberg false discovery rate (B-H FDR) of 0.20, a cutoff that is in line with a hypothesis-generating study, and the preference for some false positive findings in exchange for a larger list of potentially interesting true findings (Efron, 2010). To assess the effects of sex on miRNA levels, data generated from the participants were re-analyzed for females (n = 28) vs. males (n = 28) and analyzed by Welch’s unpaired *t*-test with a B-H FDR of 0.20. To assess the effect of disease state and APOE status, data were analyzed within each sex using a two-way ANOVA (disease x APOE genotype) followed by a Tukey’s multiple comparisons post hoc tests (n = 7/group). Given the small number of participants in this study, a covariate analysis of disease by sex by APOE genotype was not conducted. Thus, conclusions drawn from the analysis assume no interaction between all three variables.

### MiRNA Target Prediction and Pathway Analysis

We used TargetScan 7.2 (Agarwal et al., 2015) and miRDB (Liu and Wang, 2019; Chen and Wang, 2020) to predict targets of the four CSF EV miRNAs significantly associated with AD, as these programs are widely used and frequently updated. As pathway analysis is most effective for predictions generated from a limited gene set, predicted targets were excluded if they had a Cumulative Weighted Context Score (CWCS) > −0.3 in TargetScan or a target score of <60 in miRDB. To determine whether to use a union, an intersection, or an individual target list in subsequent pathway analysis, we calculated sensitivity, specificity, and precision values using validated miRNA and target pairs from miRTarBase, an experimentally validated miRNA–target interaction database (Chou et al., 2018). The values in miRTarBase range from 0–1, with high-quality results closer to 1 (Fan and Kurgan, 2015; Oliveira et al., 2017). The union of TargetScan and miRDB showed the highest values of sensitivity, specificity, and precision with values of 0.35, 0.83, and 0.98, respectively. The use of a union between the two target prediction algorithm outputs increases the sensitivity of the targets predicted and the chance of predicting a novel target, as opposed to an intersection where only targets predicted by both algorithms are included in analysis (Fan and Kurgan, 2015; Oliveira et al., 2017). Pathway analysis was then performed on the union set of 2,319 unique targets using the ingenuity pathway analysis (IPA; QIAGEN Inc., qiagenbioinformatics.com/products/ingenuity-pathway-analysis). For this analysis, we excluded cancer-related tissues and cell lines to avoid knowledge bias toward cancer in IPA and applied a B-H FDR of 0.01.

### MISEV 2018, MIFlowCyt-EV, and EV-TRACK

The isolation and characterization of CSF EVs followed recommendations from the Minimal Information of Studies of Extracellular Vesicles 2018 (MISEV 2018), a position study from the International Society of Extracellular Vesicles (ISEV) (Thery et al., 2018). The vFC experiments followed recommendations from MIFlowCyt-EV, a framework for standardized reporting of EV flow cytometry experiments from ISEV (Welsh et al., 2020). Relevant experimental parameters can be accessed in the EV-TRACK (evtrack.org) knowledgebase (EV-Track et al., 2017).

## Results

### Study Participant Characteristics

CTL CSF was pooled and used for vFC, immunoblot, TRPS, and TEM experiments, while individual CSF samples from CTL and AD participants were used for the miRNA qPCR assays. Characteristics for the 28 AD and 28 CTL participants in this study (56 total participants) are shown in Table 1. The ratio of participants was 1:1 female to male overall, with equal numbers of females and males in the AD and CTL groups. Furthermore, the APOE-e3,3 to APOE-e3,4 ratio was 1:1 within each sex to obtain a total of eight groups (disease by sex by genotype) with n = 7/group. Participants were well matched for age at the time of lumbar puncture across all the groups with no significant differences. CTL participants were in good health with a mean MMSE (Folstein et al., 1983) score of 29.3 ± 1.3. AD patients were diagnosed with probable AD according to ADRDA-NINDS criteria (McKhann et al., 1984; McKhann et al., 2011), with a mean MMSE score of 21.5 ± 3.1. There was a significant effect of disease state on the MMSE score F_(1,48)_ = 139.8, *p* < 0.0001, but no significant interactions with disease state and sex and/or genotype.

### CSF Contains a Low Concentration of Membrane-Associated TS + EVs

**FIGURE 2:**
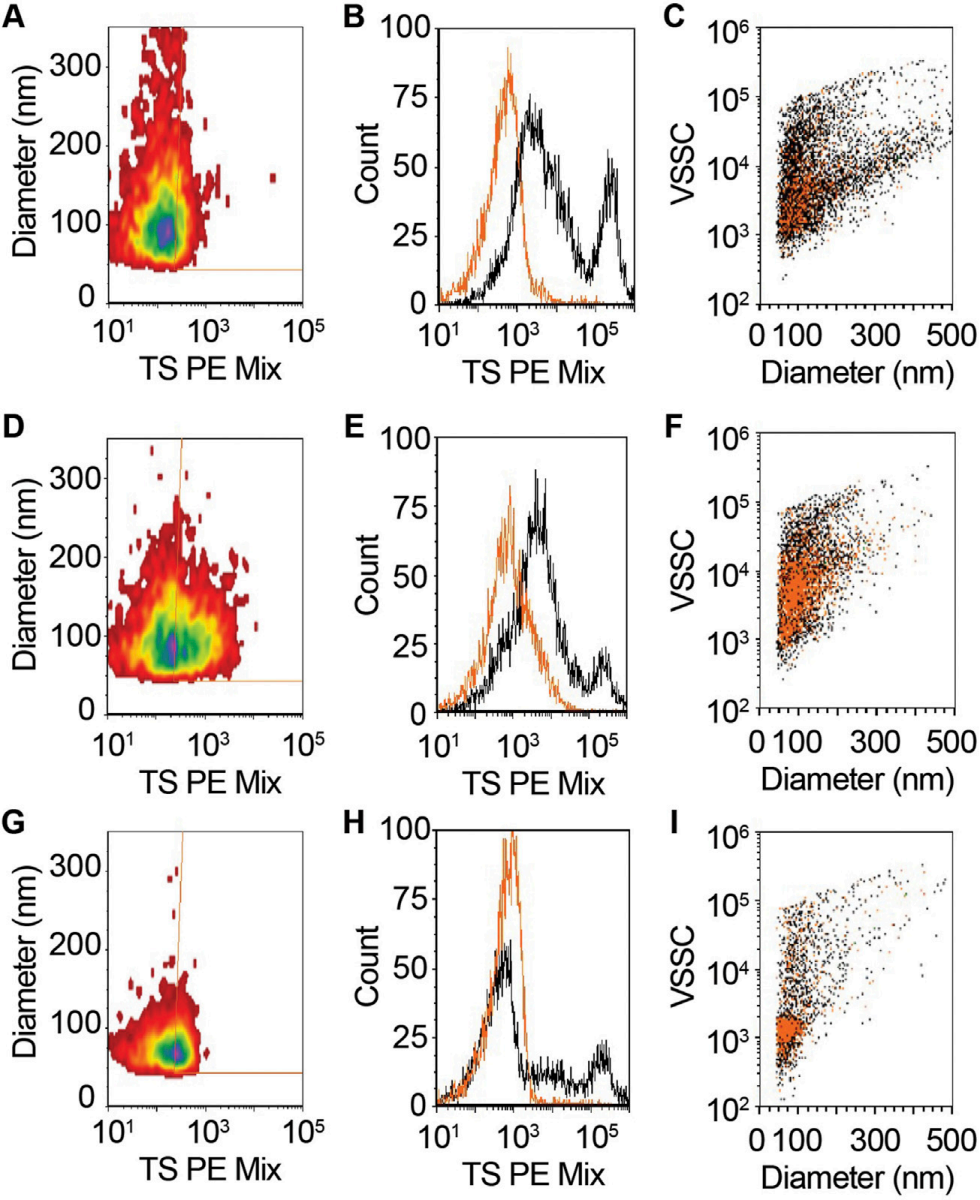
CSF contains tetraspanin (CD9, CD63, and CD81)-positive EVs. **(A–C)** Unconcentrated and **(D–F)** 25x concentrated CSF was stained with vFRed, plus a pool of PE conjugated antibodies to CD9, CD63, and CD81 (TS PE mix). **(G–I)** Treatment of stained 25x CSF with 0.1% triton X100 demonstrated the vesicular nature of the measured positive events in panels **(D–F)**. **(A,D,G)** Diameter vs. fluorescence distributions of TS + EVs. **(B,E,H)** One parameter histogram overlays of CSF with (orange) and without (black) TS + staining. **(C,F,I)** Staining events backgated onto diameter vs. violet side scatter (VSSC) for TS PE. vFRed + events are black and TS + events are orange.

To measure the size and concentration of EVs in total CSF, we performed single vFC, which uses sensitive measurements of light scatter from a fluorescent lipid probe (vFRed) and surface immunofluorescence from staining with a pool of phycoerythrin (PE)-conjugated TS antibodies (CD9, CD63, and CD81; TS PE mix). In our prior studies on plasma, we diluted the sample 150-fold prior to staining to enable detection of TS + membranous particles in the diameter range of ∼75–600 nm at mean concentration of 1.3e10/mL (Sandau et al., 2020a). However, when we performed vFC with undiluted total CSF that was stained with vFRed and the TS PE Mix, we detected TS + membranous particles just above background levels with mean size of 136 nm (range: ∼75–400 nm) and at a concentration of 1.7e6/mL (Figures 2A–C). To increase counts for the CSF assay, we subsequently used UF to concentrate 1 ml of CSF to 40 µL prior to staining with vFRed and TS PE Mix (Figure 1). The 25x concentrated CSF yielded more robust results with 1.5e7/mL TS + particles and mean particle size of 115 nm (range: ∼75–275 nm) (Figures 2D,E). Next, we detergent treated an aliquot of the stained, 25x concentrated CSF, which resulted in loss of >72% of gated events (Figures 2F,G), indicating that the majority of detected events were detergent-labile, as expected for EVs. Together, these data demonstrate that CSF contains a low concentration of TS + EVs compared to plasma.

### Small Resin Pore Size is Optimal for CSF EV Isolation by SEC

**FIGURE 3:**
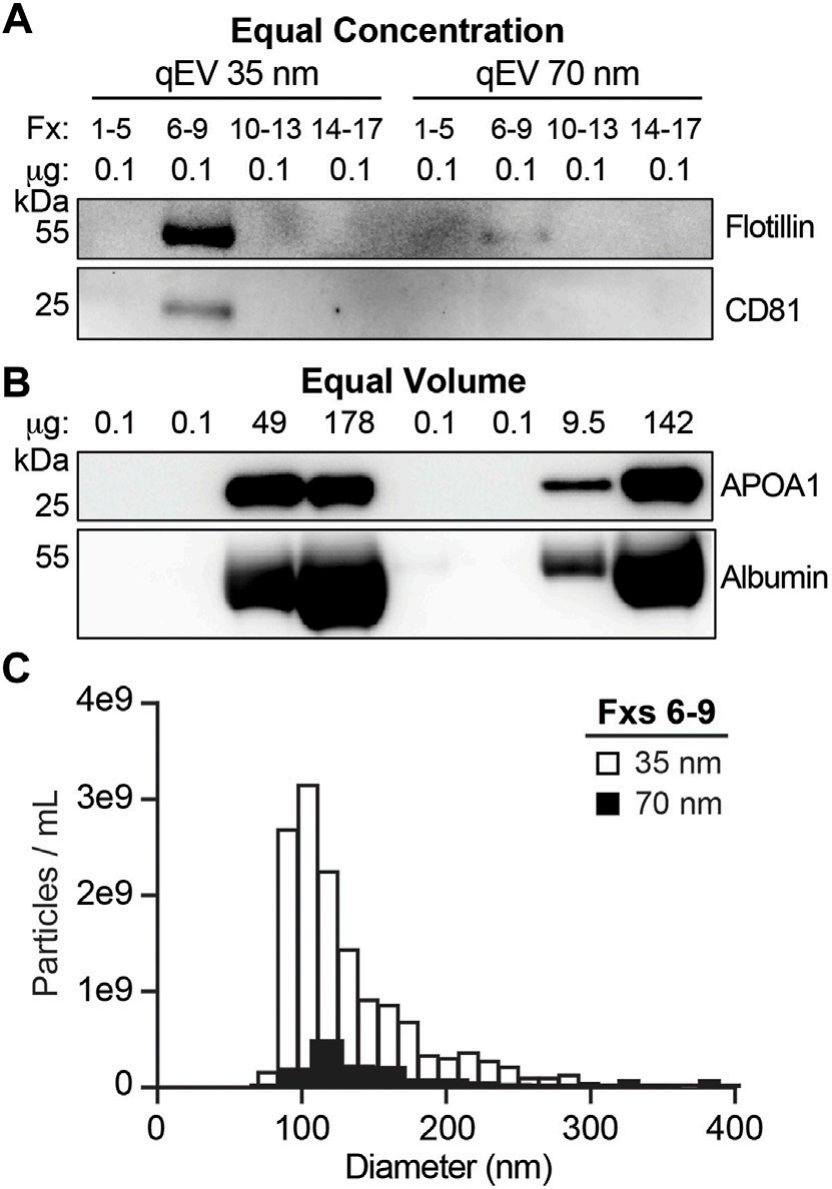
SEC qEV single 35 nm columns are optimal for separating CSF EVs. Pools of SEC fractions (Fxs): 1–5 (column void volume), 6–9, 10–13, and 14–17 were generated using either the qEV Single 35 nm or 70 nm columns. **(A)** Equal concentration loading of protein lysate (0.1 µg) from SEC pools immunoblotted for flotillin and CD81. **(B)** Equal volume loading of protein lysate (37 µL) from SEC pools immunoblotted for APOA1 and albumin. **(C)** Size and concentration histograms of Fxs 6–9 generated using either the qEV Single 35 nm (white) or 70 nm (black) columns acquired by tunable resistive pulse sensing (TRPS).

To isolate EVs from human CSF, we first performed a series of SEC protocol refinement experiments. Using CSF pooled from multiple CTL samples, we tested 35 and 70 nm SEC columns for EV enrichment as indexed by the presence of established EV protein markers (Figure 3A) and depletion of vesicle-free lipoproteins and proteins (Figure 3B). To overcome the limitations of the low concentration of EVs in total CSF (Figure 2), we started with 5 ml of CSF that was concentrated by UF to 150 µL prior to fractionating the samples into SEC pools. We collected the void volume of the column (Fxs 1–5), Fxs 6–9, 10–13, and 14–17 (Figure 1). EVs are expected to elute in Fxs 6–9 based on the manufacturer’s recommendations and our prior studies using human plasma (Sandau et al., 2020a). The SEC pools were subsequently concentrated by UF to 105 µL then assayed by immunoblot. Gels loaded by equal concentration of protein (0.1 µg) showed a strong enrichment of flotillin and CD81 in Fxs 6–9 when using the 35 nm columns. In comparison, trace amounts of flotillin were detected in Fxs 6–9 of the 70 nm columns and CD81 was undetectable (Figure 3A). Equal volume loading of concentrated SEC pools demonstrated that both the 35 and 70 nm columns effectively depleted albumin and APOA1 (vesicle free-protein and lipoprotein, respectively) from Fxs 6–9 (Figure 3B). We next used CSF fractionated by SEC on 35 and 70 nm columns to obtain size and concentration measurements by TRPS (Figure 1). The void volume for the 35 and 70 nm columns both had <10 particles of the expected size that were detected during the entirety of the maximum recoding time of 10 min (data not shown). Fxs 6–9 from the 35 nm column had 1.45e10 particles/mL with a mean size of 138 nm (range: 76–620 nm), while the 70 nm column had 1.54e9 particles/mL with a mean size of 151 nm (range: 86–360 nm) (Figure 3C). The increased abundance of smaller-sized particles obtained in Fxs 6–9 of the 35 nm column is in agreement with the immunoblot results. Together, these results demonstrate that the 35 nm SEC column is optimal for CSF EV enrichment.

### Isolated CSF EVs Have a Lipid Membrane and are Enriched for Markers of the Endolysosomal Pathway, MVs, and Astrocyte-Derived EVs

**FIGURE 4:**
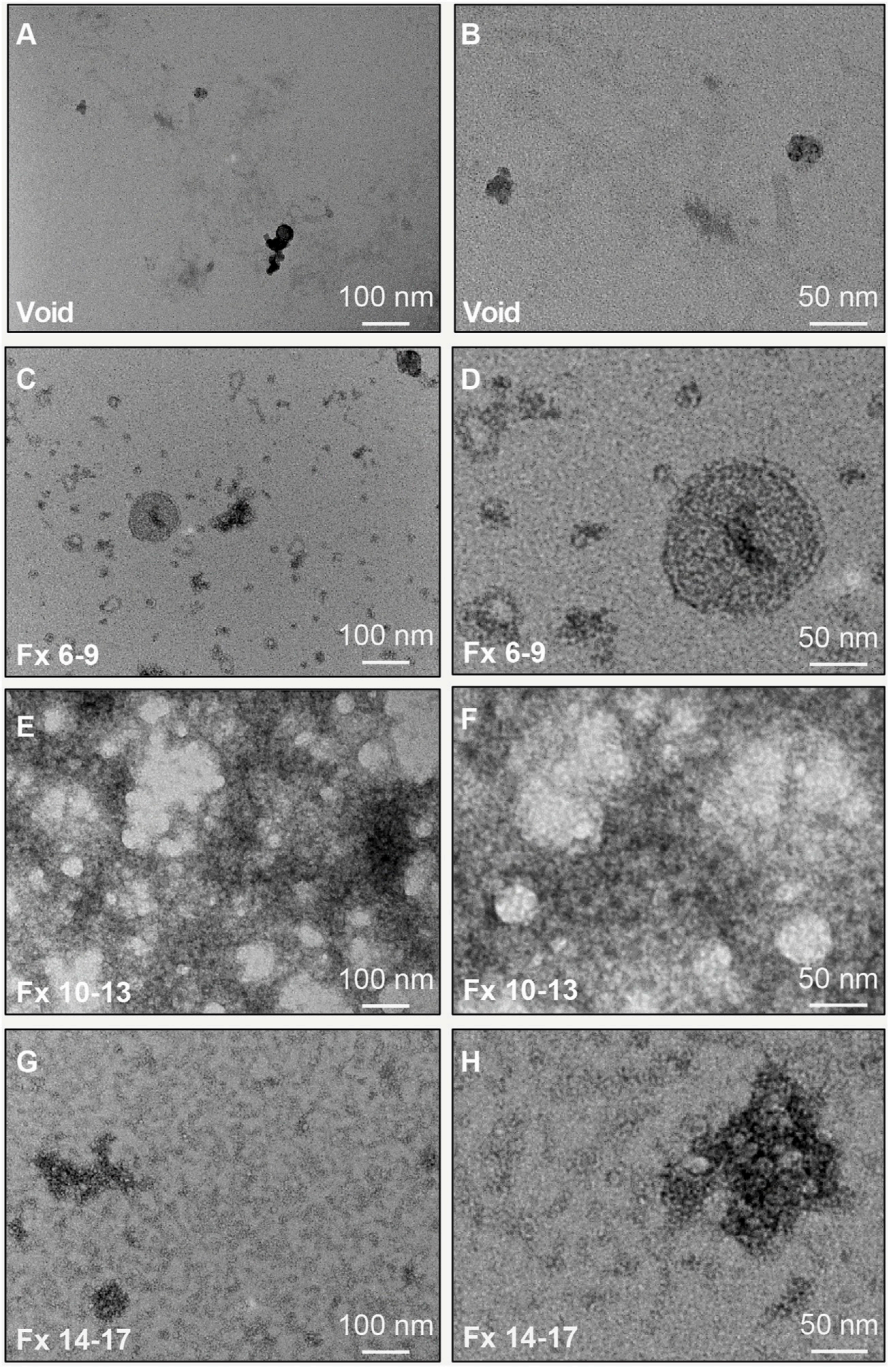
TEM characterization of CSF EVs isolated by SEC. Representative TEM images of pools of SEC fractions (Fxs): 1–5 (**(A,B)**; column void volume), 6–9 **(C,D)**, 10–13 **(E,F)**, and 14–17 **(G,H)**. Note: Fxs 6–9 show membrane bound vesicles at a size range of ∼30–100 nm, with depletion of proteins and lipoproteins, which are present in Fxs 10–13 and 14–17. Panels **(A,C,E,G)** are wide field of view (scale bars = 100 nm) and panels **(B,D,F,H)** are close up views (scale bars = 50 nm).

**FIGURE 5:**
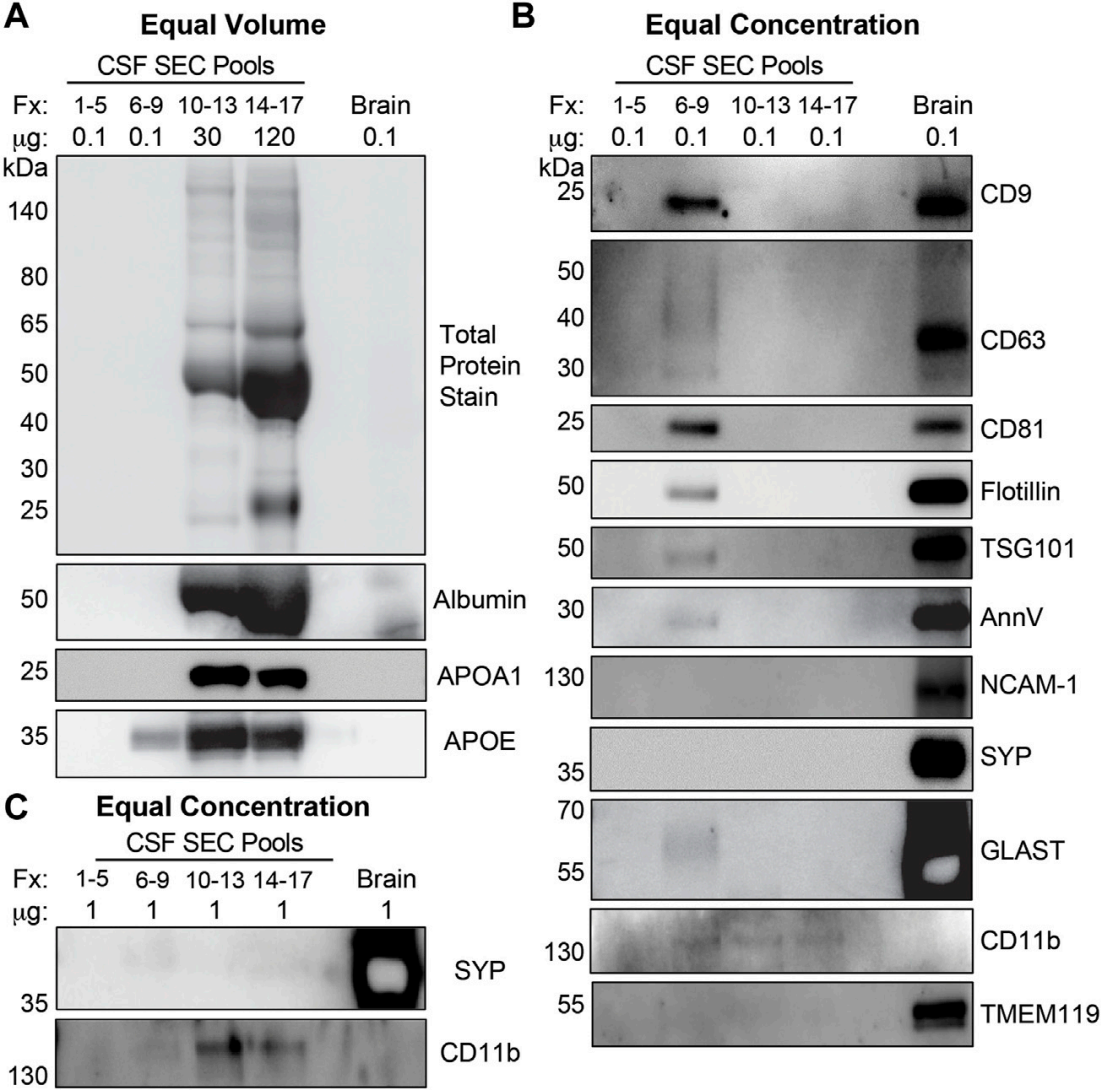
CSF EVs isolated by SEC are enriched for exosome and MV markers. **(A)** Equal volume loading of protein lysate (37 µL) from pools of SEC Fractions (Fxs): 1–5 (column void volume), 6–9, 10–13, and 14–17 stained for total protein, and immunoblotted for albumin, APOA1, and APOE. **(B)** Equal concentration loading of protein lysate (0.1 µg) from pools of Fxs 1–5, 6–9, 10–13, and 14–17 immunoblotted for CD9, CD63, CD81, flotillin, TSG101, AnnV, SYP, NCAM-1, GLAST, CD11b, and TMEM119. **(C)** Equal concentration loading of (1 µg) from pools of Fxs 1–5, 6–9, 10–13, and 14–17 immunoblotted for SYP and CD11b. Postmortem human cerebral cortex protein lysate (0.1 µg and 1 µg) was run as a positive control for each gel (**(A-C)**: Brain).

We further characterized CSF fractionated using the 35 nm SEC columns by TEM and immunoblots (Figure 1). Figure 4 shows representative wide and narrow field of view TEM images of the SEC pools. The images revealed that the void contained a small amount of diffuse material that ranges in size from 30–100 nm (Figures 4A,B). In line with our TRPS results, EVs with a lipid membrane of the expected size range for exosomes (∼40–150 nm) or small MVs (∼150–1,000) were detectable in Fxs 6–9. Furthermore, there was a greater proportion of smaller nanoparticles (<40 nm), which are the expected size range for exomeres (∼30–50 nm), supermeres (∼25–30 nm), or vesicle-free lipoproteins (∼5–35 nm) (Zhang et al., 2018; Zipkin, 2019; Zhang et al., 2021). Fxs 6–9 showed negligible levels of background protein, as expected for SEC isolates (Figures 4C,D). TEM images from Fxs 10–13 and 14–17 showed minimal numbers of EVs discernible by the presence of lipid membrane. However, there was a high degree of background, consistent with increasing amounts of vesicle-free lipoproteins and proteins that elute later (Figures 4E–H). Next, we used total protein stain and immunoblots to show a depletion of bulk proteins, albumin, and APOA1 when SEC pools were loaded by equal volume (Figure 5A). We also found that while a majority of APOE was present in Fxs 10–13 and 14–17, APOE was also detectable in the Fxs 6–9 (Figure 5A). Equal concentration loading of the SEC pools showed an enrichment of multiple EV protein markers that are associated with the endolysosomal pathway including CD9, CD63, CD81, flotillin, and TSG101 (Figure 5B) (Jeppesen et al., 2019). Furthermore, we identified an enrichment of AnnV (Figure 5B), which is associated with MVs and/or apoptotic bodies (Cocucci et al., 2009; Gouwens et al., 2018; Jeppesen et al., 2019). We next sought to determine if putative markers for brain-derived EVs were enriched in the CSF SEC pools. After loading 0.1 µg of CSF SEC Fx protein lysate, we detected an enrichment of GLAST, which is a marker for astrocyte-derived EVs (Figure 5B) (You et al., 2020). However, we were unable to detect NCAM-1 and SYP, which are markers for neuronal-derived EVs; as well as TMEM119, which is a microglia-derived EV marker (Figure 5B) (Togashi et al., 2009; Satoh et al., 2016; Sathe et al., 2019; Vukojevic et al., 2020; Cohn et al., 2021; Crews et al., 2021; Ruan et al., 2021; You et al., 2022). We also performed an immunoblot assay using 0.1 µg protein for CD11b, which is another marker for microglia-derived EVs (Gu et al., 2021), and detected faint bands in Fxs 6–9, 10–13, and 14–17 (Figure 5B). To further assess whether low concentrations of neuron- and/or microglial-derived EVs are present in CSF, we performed additional immunoblots with 1.0 µg of protein and probed for SYP and CD11b (Figure 5C). Despite a 10x increase in the amount of protein loaded, we were still unable to detect SYP in any of the CSF Fxs (Figure 5C). However, we did detect CD11b in Fxs 10–13 and 14–17, but just a faint CD11b band in Fxs 6–9 (Figure 5C). Together, these data support that using a combination of UF and SEC enriches for CSF EVs that contain markers for glial-derived EVs. However, we cannot definitively state that Fxs 6–9 are void of vesicle free lipoproteins and/or proteins based on the presence of APOE in these Fxs (Figure 5A).

### SEC Fractions Enriched for CSF EVs Contain the Greatest Number of MiRNAs

**FIGURE 6:**
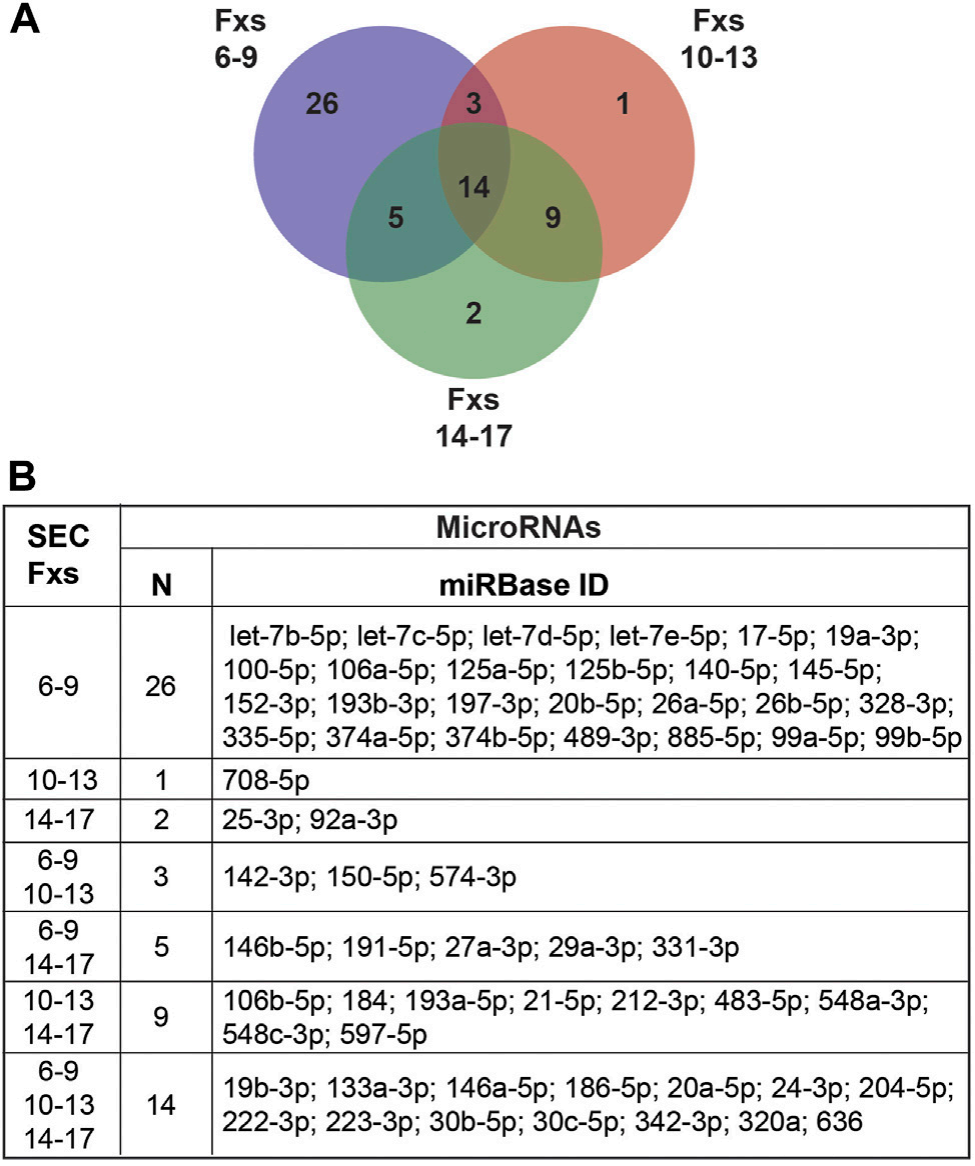
MiRNA expression in pooled CSF SEC fractions. CSF from CTLs (n = 4) was fractionated by SEC, then combined into three pools (Fxs 6–9, 10–13, and 14–17) and assayed for miRNA expression. **(A)** Venn diagram of the number of miRNAs expressed in each individual pool and those in common between the SEC pools. **(B)** Table listing the miRNAs unique to or shared between the pools. Inclusion criteria for miRNAs were expression in at least two of the four participant samples.

Considering that extracellular miRNAs are associated with exosomes, MVs, apoptotic bodies, RNA binding proteins, and high-density lipoproteins (Lasser, 2019; Mori et al., 2019), we sought to assess the miRNA profile of CSF fractionated by SEC. We concentrated 500 µL of CSF (n = 4) to 150 µL by UF; performed SEC to collect pools of Fxs 6–9, 10–13, and 14–17; and assayed miRNAs previously shown to be expressed in CSF and miRNAs validated as candidate biomarkers for AD (Wang et al., 2017; Wiedrick et al., 2019). The experiment was performed with CTL CSF balanced for sex and APOE genotype (n = 4): female APOE-e3,3, female APOE-e3,4, male APOE-e3,3, and male APOE-e3,4 (Table 1). MiRNAs were excluded from the analysis if they were not expressed in at least two of the four participants. In total, Fxs 6–9, which is enriched for CSF EVs, expressed the greatest number of miRNAs (48 total), compared to Fxs 10–13 (27 total) and 14–17 (30 total) (Figure 6). Furthermore, 26 of the 48 miRNAs that were expressed in the CSF EVs (Fxs 6–9) were not detectable in the other SEC pools, while only a few miRNAs were exclusively expressed in Fxs 10–13 and 14–17. Of the 48 miRNAs expressed in Fxs 6–9, 12 were our candidate AD biomarkers (miR-125-5p, -140-5p, -142-3p, -145-5p, -146a-5p, -146b-5p, -19b-3p, -223-3p, -24-3p, -29a-3p, -328-3p, and -331-3p) (Wiedrick et al., 2019). Together, these data demonstrate that a majority of the miRNAs expressed in CSF, including AD biomarkers, are detectable in SEC fractions enriched for EVs.

### MiRNA Cargo in AD CSF EVs is Altered and Predicts Gene Targets Relevant to AD Pathophysiology

**FIGURE 7:**
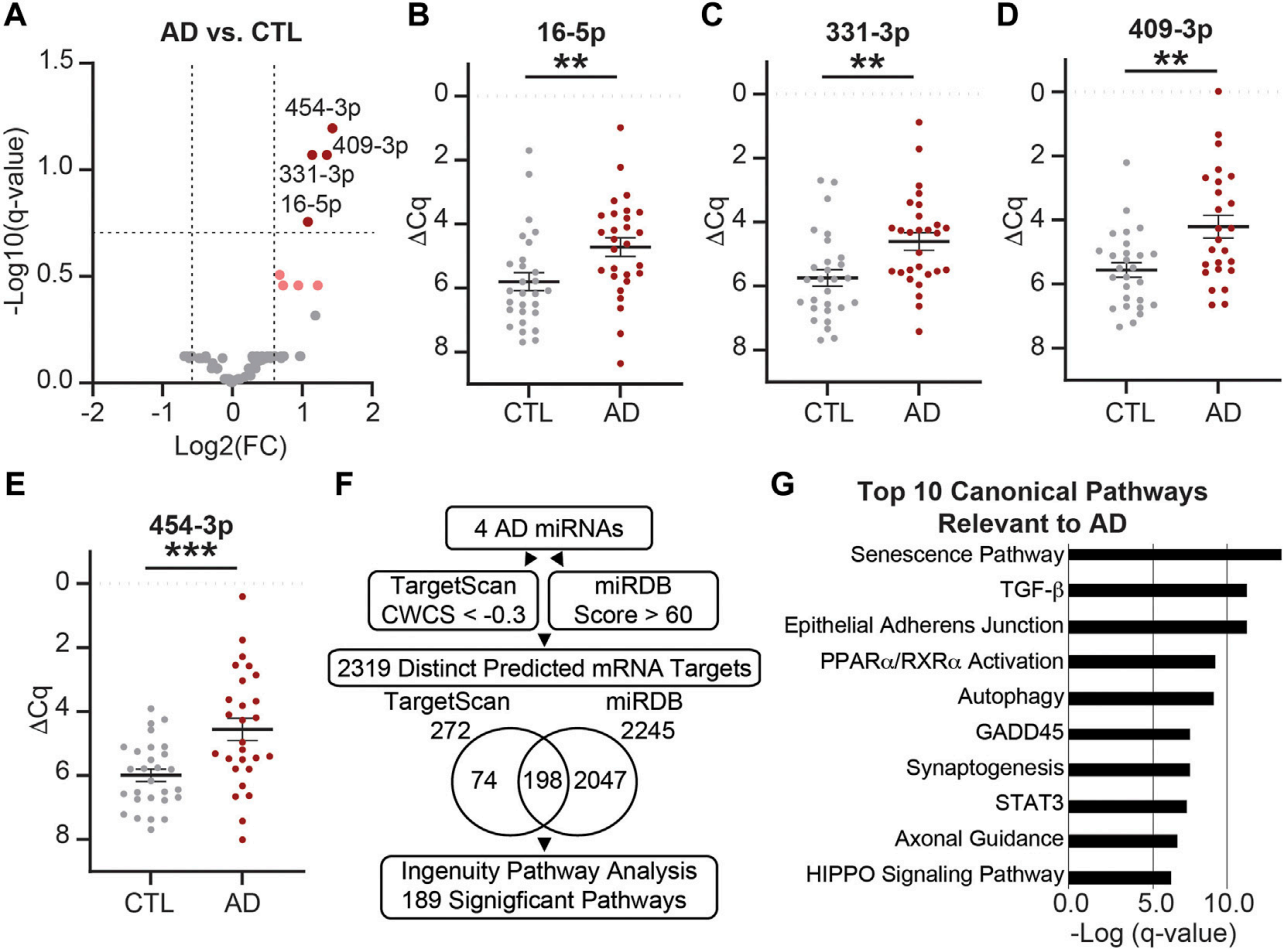
MiRNAs are differentially expressed in AD vs. CTL CSF EVs. **(A)** Volcano plot shows fold change (FC) of 71 miRNAs expressed in AD CSF EVs (n = 28) vs. CTL CSF EVs (n = 28). The vertical dashed lines correspond to 1.5 FC, and the horizontal dashed line designates the cutoff for statistically significant miRNAs (Welch’s unpaired *t*-test; *p*-value<0.011 based on Benjamini–Hochberg false discovery rate (B-H FDR) 0.20). The miRNAs designated by dark red circles are significantly increased by at least 1.5 FC in AD. MiRNAs in pink have a 1.5 FC in expression with p-value <0.05, but are below the B-H FDR threshold. **(B–E)** Normalized Cq (ΔCq) values for the four miRNAs that are significantly increased in AD vs. CTL. Data shown as the mean ± SEM and analyzed using Welch’s unpaired t-tests, **p < 0.01 and ***p < 0.001. **(F)** Workflow of the target prediction analysis for the four significant miRNAs: miR-16-5p, -331-3p, -409-3p, and -454-3p. MiRNAs were queried using TargetScan 7.2 with a CWCS of < −0.3 (272 MiRNAs total) and miRDB with a target score >60 (2,245 MiRNAs total). The Venn diagram shows the overlap in miRNA targets predicted by TargetScan 7.2 and miRDB. Of the 2,319 predicted mRNA targets (74 from TargetScan +2047 from miRDB +198 in common to both), we identified 189 significant pathways (B-H FDR 0.01). **(G)** Top 10 canonical pathways predicted by the mRNA targets, all of which are relevant to AD. Note all 10 pathways were in the top 17 of all 189 significant pathways, and senescence, TGF-β signaling, and epithelial adherens junction signaling pathways were the top three overall.

Next, we assessed the impact of AD on the miRNA cargo of CSF EVs. MiRNAs were assayed in CSF Fxs 6–9 from eight biological groups that included AD vs. CTL, female vs. male, and APOE-e3,3 vs. APOE-e3,4 participants (Table 1, n = 56 total). We identified 71 miRNAs expressed in the AD (n = 28) and/or CTL (n = 28) participant samples (Figure 7A). Of these four miRNAs were (miR-16-5p, -331-3p, -409-3p, and -454-3p) significantly increased by at least a 1.5-fold in AD relative to CTL (Figures 7A–E and Supplementary Table S3), two of which we previously identified and validated as candidate AD biomarkers (miR-16-5p and -331-3p) (Wiedrick et al., 2019). There were also four miRNAs (let-7d-5p, miR-100-5p, -374a-3p, and -378e) that increased by at least a 1.5-fold change in expression with *p* < 0.05 but were not considered significant based on the FDR (Figure 7A, pink). Next, we used the four significant miRNAs (miR-16-5p, -331-3p, -409-3p, and -454-3p) in our established target prediction pipeline using TargetScan 7.2 (Agarwal et al., 2015) and miRDB (Liu and Wang, 2019; Chen and Wang, 2020) as depicted in Figure 7F. Target prediction returned a total of 272 mRNAs by TargetScan (Supplementary Table S4) and 2,245 mRNAs by miRDB (Supplementary Table S5). Of these, 198 targets were predicted by both TargetScan and miRDB. Thus, the total number of unique predicted mRNA targets is 2,319, which were used in IPA (Qiagen) to identify pathways that have a significant overrepresentation of the predicted targets and potentially regulated by the miRNAs. IPA identified 189 significant canonical pathways (Supplementary Table S6). Of these, 10 of the top 17 pathways are known to be relevant to AD, including the top three overall pathways: senescence, TGF-β signaling, and epithelial adherens junction signaling (Figure 7G) (Caraci et al., 2018; Pearson et al., 2020; Saez-Atienzar and Masliah, 2020).

### CSF EV MiRNA Cargo is Affected by Sex and APOE Genotype

**FIGURE 8:**
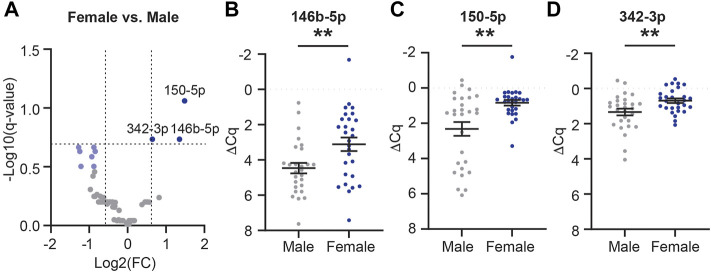
MiRNAs are differentially expressed in female vs. male CSF EVs. **(A)** Volcano plot shows fold change (FC) of 71 miRNAs expressed in CSF EVs in females (n = 28) vs. males (n = 28). The vertical dashed lines correspond to 1.5 FC, and the horizontal dashed line designates the cutoff for statistically significant miRNAs (Welch’s unpaired *t*-test; *p*-value<0.011 based on Benjamini–Hochberg false discovery rate (B-H FDR) 0.20). The miRNAs designated by dark blue circles are significantly increased by at least 1.5 FC in females. MiRNAs in light blue have a 1.5 FC in expression with p-value <0.05 but are below the B-H FDR threshold. **(B–D)** Normalized Cq (ΔCq) values for the three miRNAs that are significantly increased in females vs. males. Data shown as the mean ± SEM and analyzed using Welch’s unpaired t-tests, **p < 0.01 and ***p < 0.001.

Considering that female APOE-e4 carriers are more likely to progress from MCI to AD, develop AD more frequently than age-matched males, and have more brain atrophy and memory loss (Fleisher et al., 2008; Altmann et al., 2014; Sampedro et al., 2015), we next sought to determine if these biological factors impact CSF EV miRNA cargo. First, we assessed the effect of sex on CSF EV miRNAs, independent of AD and APOE genotype. We identified 71 miRNAs expressed in female (n = 28) and/or male (n = 28) participant samples (Figure 8A). Of these, three miRNAs (miR-146b-5p, -150-5p, and -342-3p) were significantly increased by at least 1.5-fold in females relative to males (Figures 8A–D and Supplementary Table S3), and we had previously identified miR-146b-5p as a candidate AD biomarker (Wiedrick et al., 2019). There were also seven miRNAs (miR-19b-3p, -188-5p, -223-3p, -320c, -320d, -483-5p, and -92a-3p) that were decreased by at least a 1.5-fold in females relative to males and *p* < 0.05 but were not considered statistically significant based on the FDR (Figure 8A, light blue).

**FIGURE 9:**
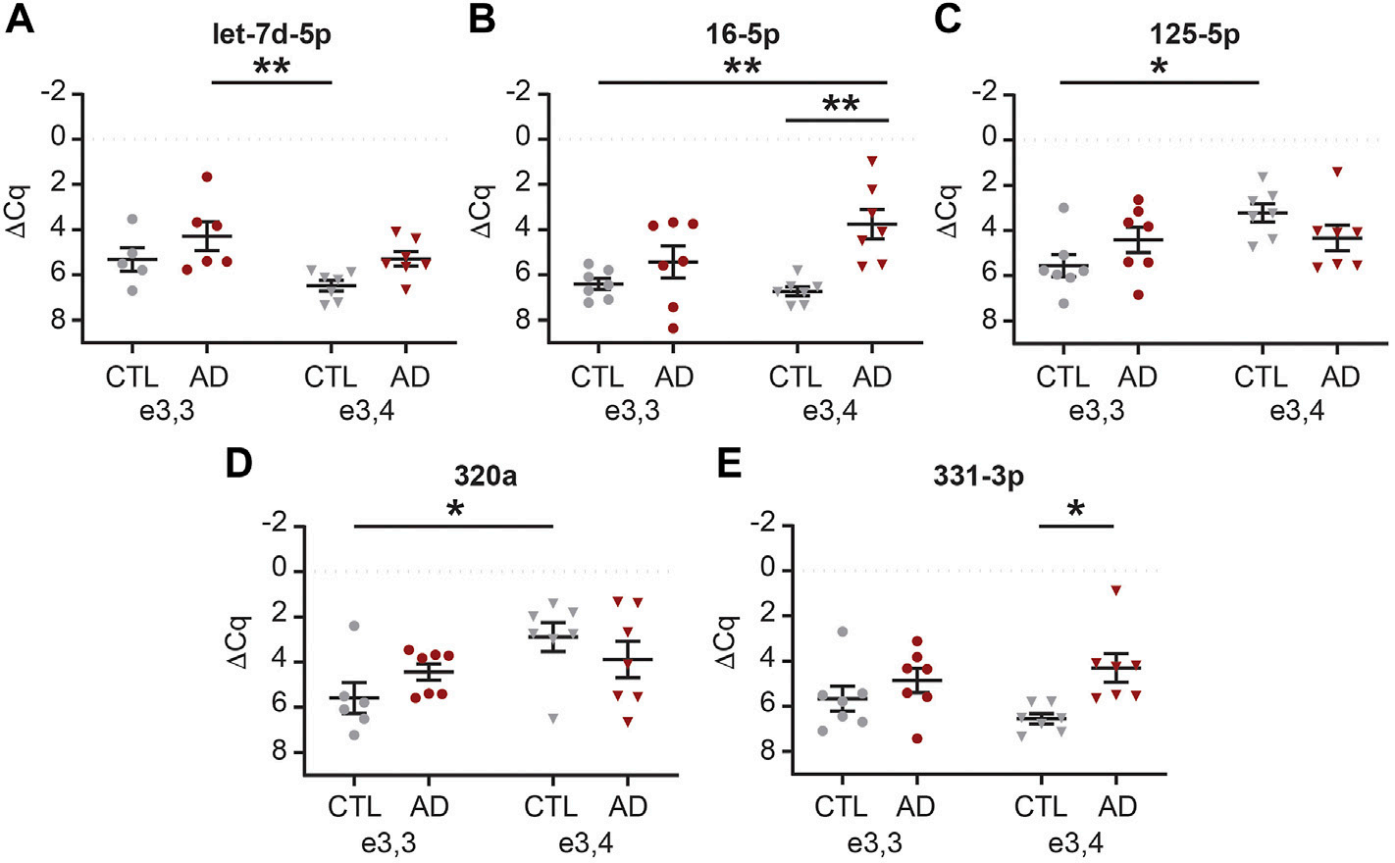
MiRNAs are differentially expressed based on APOE status and disease state in female CSF EVs. Normalized Cq (ΔCq) values for five miRNAs that demonstrate a significant effect of APOE genotype on expression levels within AD and/or CTL female CSF EVs. **(A)** Let-7d-5p was significantly increased in AD APOE-e3,3 vs. CTL APOE-e3,4. **(B)** miR-16-5p was significantly increased in AD APOE-e3,4 vs. CTL APOE-e3,3 and -e3,4. **(C)** miR-125b-5p, and **(D)** miR-320a were significantly increased in CTL APOE-e3,4 vs. CTL APOE-e3,3. **(E)** miR-331-3p was significantly increased in AD APOE-e3,4 vs. CTL APOE-e3,4. Data shown as the mean ± SEM and analyzed by two-way ANOVA followed by Tukey’s multiple comparisons post hoc tests, *p < 0.05 and **p < 0.01.

**FIGURE 10:**
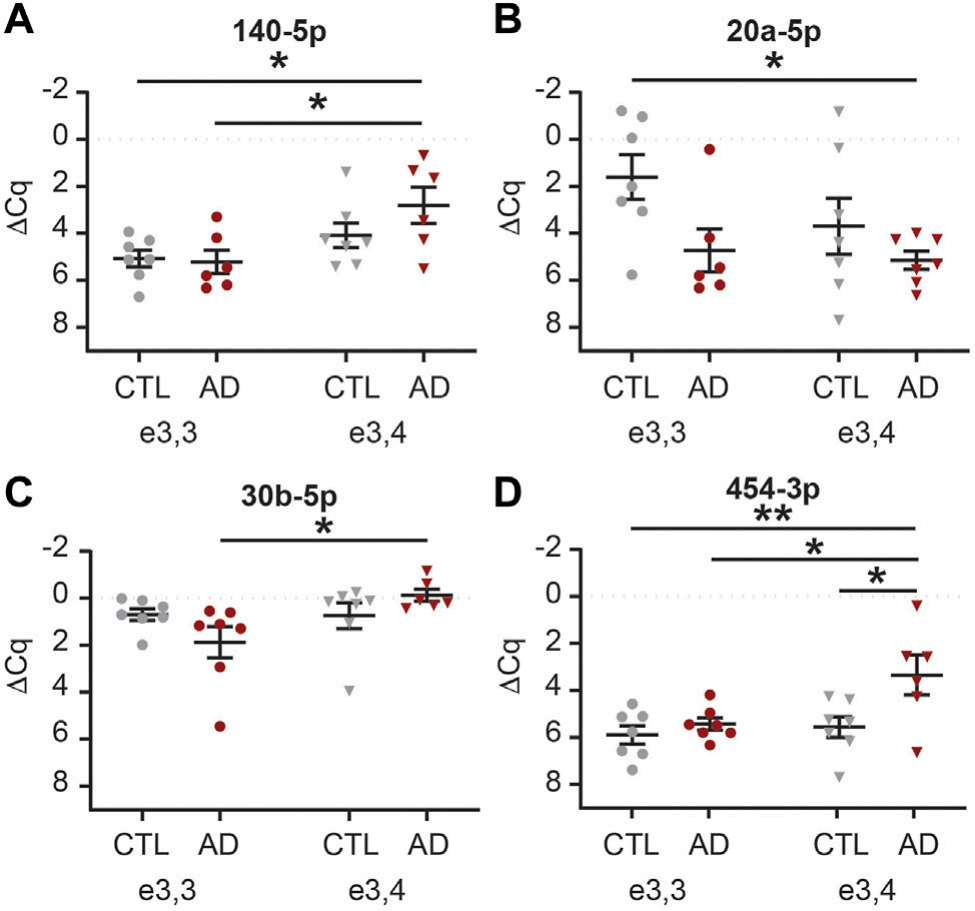
MiRNAs are differentially expressed based on APOE status and disease state in male CSF EVs. Normalized Cq (ΔCq) values for four miRNAs that demonstrate a significant effect of APOE genotype on expression levels within AD and/or CTL male CSF EVs. **(A)** miR-140-5p was significantly increased in AD APOE-e3,4 vs. AD and CTL APOE-e3,3. **(B)** miR-20a-5p was significantly decreased in AD APOE-e3,4 vs. CTL APOE-e3,3. **(C)** miR-30b-5p was significantly increased in AD APOE-e3,4 vs. AD APOE-e3,3. **(D)** miR-454-3p was significantly increased in AD APOE-e3,4 vs. all other groups. Data shown as the mean ± SEM and analyzed by two-way ANOVA followed by Tukey’s multiple comparisons post hoc tests, *p < 0.05 and **p < 0.01.

We next sought to determine if there was an interaction between AD and APOE-e4 status within a sex. First, in females, we analyzed the 71 CSF EV miRNAs for an effect of disease state and/or genotype status and identified 16 significant miRNAs (Supplementary Table S7). Of these, multiple comparisons testing identified five miRNAs with a significant group effect (Figure 9). Let-7d-5p was increased in AD APOE-e3,3 females vs. CTL APOE-e3/e4 females (Figure 9A). miR-16-5p was increased in AD APOE-e3,4 females vs. CTL APOE-e3,3 and -e3,4 females (Figure 9B). miR-125b-5p and -320a were both increased in CTL APOE-e3,4. CTL APOE-e3,3 (Figures 9C,D). miR-331-3p was increased in AD APOE-e3,4 vs. CTL APOE-e3,4 (Figure 9E). Of these five miRNAs, we previously identified three (miR-16-5p, -125b-5p, and -331-3p) as candidate AD biomarkers (Wiedrick et al., 2019). Next, our analysis of the 71 CSF EV miRNA levels in males identified five miRNAs that had a significant effect of disease state and/or genotype status (Supplementary Table S7). Of these, multiple comparisons testing identified four miRNAs with a significant change in expression (Figure 10). miR-140-5p was increased in AD APOE-e3,4 males vs. AD and CTL APOE-e3,3 males (Figure 10A). miR-20a-5p was decreased in AD APOE-e3,4 males vs. CTL APOE-e3,3 males (Figure 10B). miR-30b-5p was increased in AD APOE-e3,4 males vs. AD APOE-e3,3 males (Figure 10C). miR-454-3p was increased in AD APOE-e3,4 males vs. all other groups (Figure 10D). Of these four miRNAs, we previously identified miR-140-5p as a candidate AD biomarker (Wiedrick et al., 2019). Together, these data demonstrate that biological factors such as sex and genotype can impact the levels of miRNA cargo of CSF EVs. Note that due to the low sample size we were unable to perform a statistical analysis to assess the effect of AD by sex by APOE genotype.

## Discussion

The primary goal of this study was to determine the effect of AD and its biological risk factors on the levels of miRNA in human CSF EVs. We used a combined approach of UF plus SEC to isolate EVs from the CSF of AD and CTL participants that were balanced for sex and APOE genotype (Table 1). In line with prior reports that demonstrated an effect of AD on the CSF EV miRNAs (Liu et al., 2014; Gui et al., 2015; Riancho et al., 2017; McKeever et al., 2018; Schneider et al., 2018; Jain et al., 2019; Kumar and Reddy, 2021; Tan et al., 2021), we identified that miR-16-5p, -331-3p, -409-3p, and -454-3p had a significant 1.5 fold increase in expression in AD vs. CTL (Figure 7). Next, our pathway analysis of these four miRNAs altered in AD CSF EVs revealed that the top canonical pathways included senescence and autophagy, which are affected by EVs in AD (Misawa et al., 2020; Vassileff et al., 2020). We then investigated the effect of sex and APOE genotype status on CSF EV miRNAs and found an increase in miR-146b-5p, -150-5p, and -342-3p in females relative to males (Figure 8). We also found that APOE genotype status affects different subsets of CSF EV miRNAs in females vs. males (Figures 9, 10). Together, our data demonstrate that the miRNA cargo of CSF EVs is informative for AD and sensitive to both sex and APOE-e4 genotype. They also reveal that biological factors associated with AD risk impact the EV miRNA cargo and should be taken into consideration for mechanistic and biomarker studies.

Over the past decade, there has been great interest in exploring the utility of circulating RNAs, especially miRNAs, as biomarkers for human disease (Quinn et al., 2015). Through the Extracellular RNA Communication Consortium (ERCC), RNA biomarkers across a spectrum of biofluids and disease states have been identified including, but not limited to, glioblastoma, vascular inflammation and cardiometabolic health, and multiple sclerosis (Ainsztein et al., 2015; Regev et al., 2016; Akers et al., 2017; Shah et al., 2017a; Shah et al., 2017b; Klingenberg et al., 2017; Nonaka and Wong, 2017). In our prior ERCC studies, we focused on the utility of extracellular miRNAs as biomarkers for AD. We performed a series of discovery and validation studies to identify 25 biomarker candidates that classify AD from CTLs (Lusardi et al., 2017; Wiedrick et al., 2019). We also demonstrated that five (miR-142-3p, -146a-5p, -146b-5p, -193a-5p, and -365a-3p) of these 25 CSF miRNAs are sensitive to early-stage pathology as exemplified by MCI diagnosis (Sandau et al.). These five miRNAs jointly predicted AD with area under the curve (AUC) of 0.770 and MCI with AUC of 0.705. We further showed that while the ratio of CSF Aβ_42_:total-Tau (clinical markers for AD diagnosis (Maddalena et al., 2003)) predicted MCI with AUC of 0.758, Aβ_42_:total-Tau plus the five miRNAs improved the AUC to 0.813 (Sandau et al., 2020b). In addition, the five miRNAs plus APOE-e4 status improved classification performance for both AD and MCI relative to CTL (Sandau et al., 2020b). Aside from our studies, three (miR-142-3p, -146a-5p, and -146b-5p) of these miRNAs have been identified by others as candidate biomarkers for MCI and/or AD in total CSF (Cogswell et al., 2008; Alexandrov et al., 2012; Kiko et al., 2014; Denk et al., 2015; Nagaraj et al., 2017; Park et al., 2019). Together, these data support that CSF miRNAs carry novel information relevant to AD, even in MCI.

Extracellular miRNAs have multiple carrier types including exosomes, exomeres, supermeres, MVs, apoptotic bodies, RNA-binding proteins, and high-density lipoproteins (Vickers and Remaley, 2012; Mori et al., 2019; Zhang et al., 2021). In AD, there are disruptions in the endolysosomal pathway within both neurons and glia that affect exosome biogenesis, including RNA and protein cargo (Asai et al., 2015; Goetzl et al., 2016; Mathews and Levy, 2019; Li et al., 2020; Guo et al., 2021). EVs released from CNS cells contribute to cell-to-cell communication throughout the CNS and the periphery (Chivet et al., 2012; Dickens et al., 2017; Zhang and Yang, 2018) in normal and pathological processes (Yuyama and Igarashi, 2016; Neven et al., 2017). Thus, there is interest in exploring the molecular cargo of EVs as a biomarker for disease. However, to the best of our knowledge only a limited number of studies have assayed miRNA expression of CSF EVs in AD (Liu et al., 2014; Gui et al., 2015; Riancho et al., 2017; McKeever et al., 2018; Schneider et al., 2018; Jain et al., 2019; Kumar and Reddy, 2021; Tan et al., 2021). Furthermore, differences in EV isolation techniques can affect outcomes and render data comparison between studies more challenging.

Prior studies that investigated the miRNA cargo of CSF EVs in AD used precipitation, ultracentrifugation, or density gradient ultracentrifugation-based methods to fractionate CSF (Liu et al., 2014; Gui et al., 2015; Riancho et al., 2017; McKeever et al., 2018; Schneider et al., 2018; Jain et al., 2019; Kumar and Reddy, 2021; Tan et al., 2021). EV precipitation methods have high yields, but low specificity for isolating EVs away from vesicle-free proteins, lipoproteins, and their associated miRNAs (Furi et al., 2017; Stranska et al., 2018; Thery et al., 2018; Karttunen et al., 2019; Ter-Ovanesyan et al., 2021). Isolating EVs by ultracentrifugation results in intermediate yields and purity with potential contamination by non-vesicular macromolecules (Furi et al., 2017; Thery et al., 2018). However, density gradient ultracentrifugation fractionation of CSF results in low yield, but higher purity than other fractionation techniques (Furi et al., 2017; Thery et al., 2018). Here, we isolated EVs from CSF using a combined approach of UF and SEC, which is reported to have an intermediate yield and potential contamination with vesicle free-proteins and lipoproteins, albeit to a lesser degree than precipitation-based methods (Boing et al., 2014; Furi et al., 2017; Stranska et al., 2018; Thery et al., 2018; Ter-Ovanesyan et al., 2021). The increased yield is a key factor considering the low amount of EVs in CSF, given that the total protein content of CSF is 50–100 times lower than plasma (Yuan and Desiderio, 2005). In line with this, our vFC calculated 1.5e7/mL TS + EVs in CSF (Figure 2) vs. 1.3e10/mL TS + EVs in plasma (Sandau et al). In order to increase the yield of CSF EVs, we tested two SEC column resin sizes (35 and 70 nm). The 35 nm SEC columns showed optimal enrichment for CSF EVs based on the protein markers flotillin and CD81 as well as a 10-fold increase in the concentration of isolated particles measured by TRPS (Figure 3). These data are consistent with a recent study that also reported a smaller resin size increases SEC yield of CSF EVs (Ter-Ovanesyan et al., 2021).

While our goal was to increase yield, we also wanted to minimize vesicle-free lipoproteins and proteins, which can carry miRNAs (Vickers and Remaley, 2012; Mori et al., 2019). Our CSF EV isolation method demonstrated that SEC Fxs 6–9 were enriched for EVs (∼40–150 nm) as well as smaller nanoparticles < 40 nm based on TEM (Figure 4). These smaller particles are of the expected size for exomeres (∼30–50 nm), supermeres (∼25–30 nm), and vesicle-free lipoproteins (∼5–35 nm) (Zhang et al., 2018; Zipkin, 2019; Zhang et al., 2021). By immunoblot, Fxs 6–9 showed an enrichment of exosome protein markers associated with the vesicle membrane (CD9, CD63, and CD81) and cytosol (flotillin-1 and TSG101) (Figure 5) (Thery et al., 2018; Jeppesen et al., 2019). Fxs 6–9 were also depleted in the APOA1 lipoprotein and albumin (Figure 5), which are not-associated with EVs, but are abundant in plasma and serum and can be a source of contamination in CSF and CSF EV preparations (Thery et al., 2018; Jeppesen et al., 2019). However, while a majority of APOE was in the later fraction, Fxs 6–9 also contained the lipoprotein (Figure 5). Prior studies have shown that the protein cargo of both neuronal- and astrocyte-derived EVs includes APOE (Nikitidou et al., 2017; Zheng et al., 2018), which is also increased in the secreted EVs following Aβ_42_ treatment (Nikitidou et al., 2017). Together, our data support that using a combination of UF and SEC does enrich for CSF EVs; however, we cannot state the identity of the small nanoparticles that are also present in Fxs 6–9. Additional experiments using immunoTEM in SEC fractions with markers associated with exosomes (CD81), exomeres (Ago1/2), supermeres (ENO2, Ago1/2), and APOE are needed (Zhang et al., 2021). Furthermore, experiments that combine proteinase K treatment with immunoaffinity capture or immunoTEM are also needed to determine if APOE is cargo for EVs or associated with the EV protein corona (Toth et al., 2021).

We also used protein markers to identify the types of EVs that are present in CSF fractionated by SEC (Figure 5). Our detection of CD9, CD63, CD81, flotillin-1, and TSG101 supports the presence of exosomes in CSF, as these proteins are membrane markers for exosomes and mediators of exosome biogenesis *via* the endolysosomal pathways (Jeppesen et al., 2019). The enrichment of AnnV suggests that MVs are also present in CSF, as AnnV is a marker for MVs and/or apoptotic bodies (Cocucci et al., 2009; Gouwens et al., 2018; Jeppesen et al., 2019). While the TRPS and TEM size estimates of our CSF EV fractions (Figures 3, 4) are more in line with the enrichment of MVs (∼150–1,000 nm) as opposed to apoptotic bodies (∼500–2000 nm) (Crescitelli et al., 2013), additional experiments to rule out the presence of apoptotic bodies are needed. We also assessed the enrichment for markers of neuron- (NCAM-1 and Synaptophysin), astrocyte- (GLAST), and microglia-derived EVs (TMEM119 and CD11b) (Pouget et al., 1971; Togashi et al., 2009; Satoh et al., 2016; Sathe et al., 2019; Vukojevic et al., 2020; Zhou et al., 2020; Gu et al., 2021; Kumar et al., 2021; You et al., 2022). Analysis of 0.1 µg of pooled CTL CSF SEC fractions revealed astrocyte-derived (GLAST+) EVs, but not neuronal- (NCAM-1+ and SYP+) or microglial- (TMEM119+ and CD11b+) derived EVs. It is important to note that neuron- or microglial-derived EVs may be present in CSF at low concentrations that are below the detection limits of immunoblot using 0.1 µg of protein. Thus, to address this, we performed additional SYP and CD11b immunoblots with 1 µg of protein, which demonstrated undetectable levels of neuronal-derived EV markers, but the presence of very low levels microglial-derived EV markers. Together, these data demonstrated that performing experiments with more sensitive techniques (e.g., vFC) may be necessary to assay low abundance populations of cell-specific EVs. It is also important to note that our immunoblot assays were limited to two markers each for neuronal or microglial-EVs. Thus, additional markers may be better suited to identify neuronal (e.g., ATP1A3) or microglial (e.g., LCP1) EVs in CSF (You et al., 2022). L1CAM has been used for the enrichment of neuronal-derived EV from plasma (Winston et al., 2016; Goetzl et al., 2019; Pulliam et al., 2019; Goetzl et al., 2020; Nogueras-Ortiz et al., 2020; Anastasi et al., 2021; Cressatti et al., 2021; Saeedi et al., 2021; Yao et al., 2021). However, the use of L1CAM as a neuronal EV marker is an ongoing debate in the EV community, in part due to a recent and comprehensive study demonstrated that L1CAM in CSF is not a marker of neuronal EVs (Norman et al., 2021). Another, important limitation of our study is that the CSF EV profile is based on a pool of CTL samples and not individual participants that reflect disease state. Thus, our future experiments include comprehensive profiling of CSF EVs in both AD and CTLs to identify and assess the relative abundance of cell-type specific markers for neurons, astrocytes, and microglia by both immunoblot and vFC.

Considering that extracellular RNAs can be transported by EVs, exomeres, supermeres, lipoproteins, and RNA-binding proteins (Vickers and Remaley, 2012; Mori et al., 2019; Zhang et al., 2021) and that FXs 6**–**9 showed an enrichment of EVs and smaller nanoparticles, while Fxs 10–13 and 14–17 contained a majority of the vesicle-free lipoproteins and proteins we sought to determine the miRNA profiles of each pool (Figure 6). The largest total number miRNAs were detected in Fxs 6**–**9, including 26 miRNAs that were unique to these fractions, and 12 miRNAs that we previously identified as candidate biomarkers for AD in total CSF (miR-125b-5p, -140-5p, -142-3p, -145-5p, -146a-5p, -146b-5p, -19b-3p, -223-3p, -24-3p, -29a-3p, -328-3p, and -331-3p), with five being unique to Fxs 6–9 (miR-125b-5p, -140-5p, -145-5p, -19b-3p, and -328-3p). In comparison, we identified very few miRNAs that were unique to the fractions that contained a majority of the vesicle-free lipoproteins and proteins (Fxs 10–13 and 14–17). However, we did observe 14 miRNAs that were in common between all three pooled fractions, including five AD CSF biomarkers (miR-146a-5p, -19b-5p, -24-3p, and -224-3p). Also, there were two AD biomarkers detected only in the vesicle-free lipoprotein and protein fractions (miR-19b-3p and 193a-5p). Note that some miRNAs included in the AD, sex, and APOE analyses were excluded from this analysis (e.g., miR-16-5p) because of differential detection in AD only and not expressed in at least two of the four CTL samples used in this experiment. Together, these data demonstrate that SEC fractions, which contain EVs and small nanoparticles, also contain a majority of the miRNAs that are informative about disease state. However, miRNAs carried by lipoproteins and RNA-binding proteins also have biomarker potential. Thus, more comprehensive studies are needed to fully elucidate the effects of AD on miRNA transport in CSF.

Our analysis of CSF EV miRNA expression between AD and CTL participants identified four miRNAs that were significantly increased in AD (Figure 7). We had previously identified two (miR-16-5p and -331-3p) of these four miRNAs as biomarkers for AD using total CSF (Wiedrick et al., 2019). Two independent studies that have also shown differential expression of miR-16-5p in CSF EVs from AD participants, compared to CTLs. In contrast to our findings, both studies reported a decrease in miR-16-5p expression levels in AD CSF EVs (Gui et al., 2015; McKeever et al., 2018). The differences may be associated with participant age at time of lumbar puncture. For our study, the average age of AD participants was 73 years old, while the average ages from the other studies were 61 and 63 years old (Gui et al., 2015; McKeever et al., 2018). Together, these data suggest that miR-16-5p expression levels may be sensitive to disease stage, which is further supported by studies in human postmortem AD tissue that show increased miR-16-5p in the prefrontal cortex and hippocampus of postmortem AD brain compared to CTL (Lau et al., 2013; Muller et al., 2014). Of the other miRNAs, two that were significantly increase in AD CSF EVs (miR-409-3p and -454-3p, Figure 7) have also been shown to be differentially expressed in postmortem AD prefrontal cortex compared to CTL (Lau et al., 2013). Together, these data demonstrate that the miRNA cargo of EVs is sensitive to neuropathological effects of AD.

Our target prediction analysis (Figure 6) found that 10 of the top 17 significant pathways are involved in AD. The top pathway overall was senescence, the permanent arrest of a proliferative cell (Saez-Atienzar and Masliah, 2020). In a study using AD brains, immunostaining showed the accumulation of senescent astrocytes (Bhat et al., 2012). Additionally, astrocyte senescence has been shown to promote glutamate excitotoxicity in cortical neurons, which may lead to neurodegeneration (Limbad et al., 2020). Within the senescence pathway, we identified SMAD2 and TGFB2 as predicted gene targets of miR-409-3p and -454-3p, respectively (Supplementary Table S5). In an APP/APOE knockout mouse, inhibition of the TGFB/SMAD2 pathway activity in astrocytes has been shown to increase amyloid plaque formation and cognitive impairment (Zheng et al., 2017). This could suggest that EV miRNAs may act to suppress SMAD2 and TGFB2 translation in astrocytes and contribute to disease pathogenesis. Since our CSF EVs were enriched for GLAST and likely associated with astrocytes, the miRNAs may be part of an astrocyte-derived mechanism that drives senescence and subsequently neurodegeneration. The top 10 pathways also included autophagy, which is essential to maintain the homeostasis of neurons. In AD brains, autophagy defects attribute to accumulation of cellular waste and autophagic organelles in dystrophic neurites (Nixon et al., 2005). In neurons, autophagosomes often fuse with MVBs before depositing contents to the lysosome for degradation (Colacurcio et al., 2018). Disruptions in the fusion of autophagosomes to the MVB can cause MVB accumulation of amyloid precursor protein and the toxic C-terminal fragment resulting from its cleavage, along with reduced degradation of these products (González et al., 2017). As MVBs are also a prominent site of EV biogenesis (Abels and Breakefield, 2016), disruption of MVBs due to autophagy defects may affect both the biogenesis of EVs and their contents. In line with our target prediction, a study that profiled the protein cargo of CSF EVs from AD patients found several of the same affected pathways (Muraoka et al., 2020). A prominent AD-related protein in the autophagy pathway is PTEN (Diaz Gonzalez et al., 2021), which we identified as a potential gene target of miR-454-3p (Supplementary Tables S4, S5). Pharmacological upregulation of PTEN has been shown to induce autophagy and increase clearance of Aβ in primary neuronal cells (Wani et al., 2019). Together, these data could contribute to the discovery of novel therapeutic targets for proteins and pathways regulated by miRNAs that are involved in AD.

APOE-e4 has been shown to disrupt exosome biogenesis and decreases the production of brain EVs in aged non-AD human brain and a humanized APOE-e4 mouse model (Peng et al., 2019; Ben Khedher et al., 2021). Furthermore, APOE-positive particles produced in cultured astrocytes can transport miRNAs to neurons with astrocyte-derived APOE-e4 particles carrying fewer miRNAs compared to APOE-e3 particles (Li et al., 2021). Both APOE alleles have also been shown to regulate miRNA expression (Han et al., 2013; Sharma and Eghbali, 2014). APOE-e4 decreases expression of miR-146a in the brain of both wild-type and 5xFAD AD mice compared to APOE-e3 (Teter et al., 2016). Aside from APOE-e4, miRNA expression is also regulated by both hormonal (estradiol, progesterone, and testosterone) and genetic differences between females and males (Waltering et al., 2011; Klinge, 2012; Lam et al., 2012; Sharma and Eghbali, 2014). Hormones can regulate miRNA expression directly through gene transcription *via* ligand bound nuclear hormone receptors that bind and recruit co-activators and–repressors to gene promoter elements. In rodents, 17β-estradiol differentially regulates miRNA expression in an age-dependent manner in the hippocampus (Rao et al., 2013). Furthermore, in cultured neurons, 17β-estradiol regulates miR-125b expression and prevents Aβ-induced neurotoxicity (Micheli et al., 2016). While these studies all implicate an effect of AD, APOE genotype status, and sex on EV and/or miRNAs, there is a knowledge gap in human studies. To the best of our knowledge, aside from the study herein, eight studies have investigated miRNA expression in CSF EVs from AD participants (Liu et al., 2014; Gui et al., 2015; Riancho et al., 2017; McKeever et al., 2018; Schneider et al., 2018; Jain et al., 2019; Kumar and Reddy, 2021; Tan et al., 2021), and we are the first to investigate the effects of sex and APOE-e4 status on CSF EVs and their miRNA cargo. Considering the relatively limited number of studies that have investigated sex-dependent effects on EVs and their RNA cargo, future studies that address basic questions such as the effect of sex on EV biogenesis, circulating EV concentration, and total concentration of RNA cargo are warranted.

In summary, our studies identified that AD and two of its risk factors, sex and APOE-e4 status, impact CSF EV miRNA levels. Importantly, APOE-e4 status within each sex-altered different subsets of miRNAs. Furthermore, the miRNAs differentially expressed in AD CSF EVs predicted gene targets and pathways that are known to contribute to neurodegeneration in AD. Together, these data demonstrate the need to perform large scale studies that take into consideration key biological factors in addition to the disease state in order to more precisely define biomarker profiles for the at-risk population. Our data also demonstrate that EV miRNAs could facilitate the identification of new targets for the therapeutic treatment of AD and define how EV-derived miRNAs may render females and/or APOE-e4 carriers more prone to AD.

## Resource Identification Initiative

**Table:** 

**Antibody or Tool/Resource**	**RRID**
Albumin antibody	AB_2225785
Annexin V antibody	AB_1949660
APOA1 antibody	AB_1119020
APOE antibody	AB_2904241
CD9 antibody	AB_627213
CD11b antibody	AB_2650514
CD63 bntibody	AB_2800495
CD81 bntibody	AB_2275892
Donkey anti-rabbit IgG HRP antibody	AB_10015282
Donkey anti-goat IgG HRP antibody	AB_2340390
Donkey anti-mouse IgG HRP antibody	AB_2340770
Flotillin antibody	AB_11156367
GLAST antibody	AB_2190597
NCAM-1 antibody	AB_2904242
Synaptophysin antibody	AB_2799098
TMEM119 antibody	AB_2882272
TSG101 antibody	AB_10974262
Bio-Rad ChemiDoc Touch	SCR_021693
Bio-Rad T100 PCR	SCR_021921
CytoFlex S	SCR_019627
ExpressionSuite software	SCR_021095
FCS Express software	SCR_016431
GraphPad Prism software	SCR_002798
Ingenuity Pathway analysis	SCR_008653
IZON Control Suite software	SCR_021922
IZON qNANO Gold	SCR_021923
miRBase	SCR_003152
miRDB	SCR_010848
Orbitor RS2 Microplate Robot	SCR_020721
QuantStudio™ 12K Flex real-time PCR system	SCR_021098
QuantStudio™ 12K Flex real-time PCR software	SCR_021096
TargetScan v.7.2	SCR_010845
OHSU Electron Microscopy Core	SCR_009969
OHSU Flow Cytometry Shared Resource	SCR_009974
OHSU Gene Profiling Shared Resource Core Laboratory	SCR_009975

## Data Availability

The authors acknowledge that the data presented in this study must be deposited and made publicly available in an acceptable repository, prior to publication. Frontiers cannot accept a article that does not adhere to our open data policies.
