# Supplementary figures and images for: Differential Effects of APOE Genotype on MicroRNA Cargo of Cerebrospinal Fluid Extracellular Vesicles in Females With Alzheimer’s Disease Compared to Males
Source: Front Cell Dev Biol. 2022 Apr 27;10:864022. doi: 10.3389/fcell.2022.864022 (PMC9092217; doi:10.3389/fcell.2022.864022)

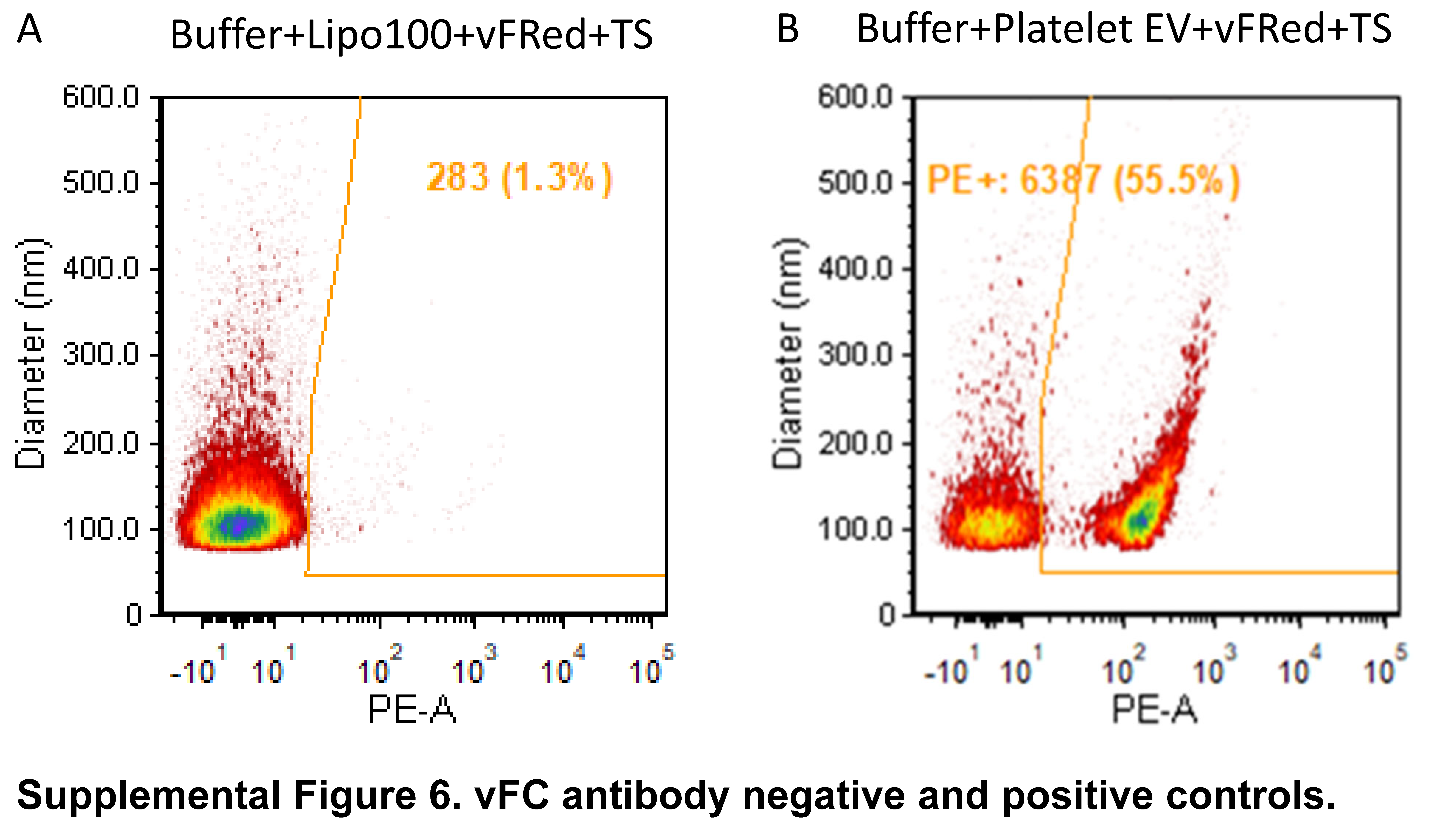

Supplement: Supplementary file 2 [file Image6.TIF]

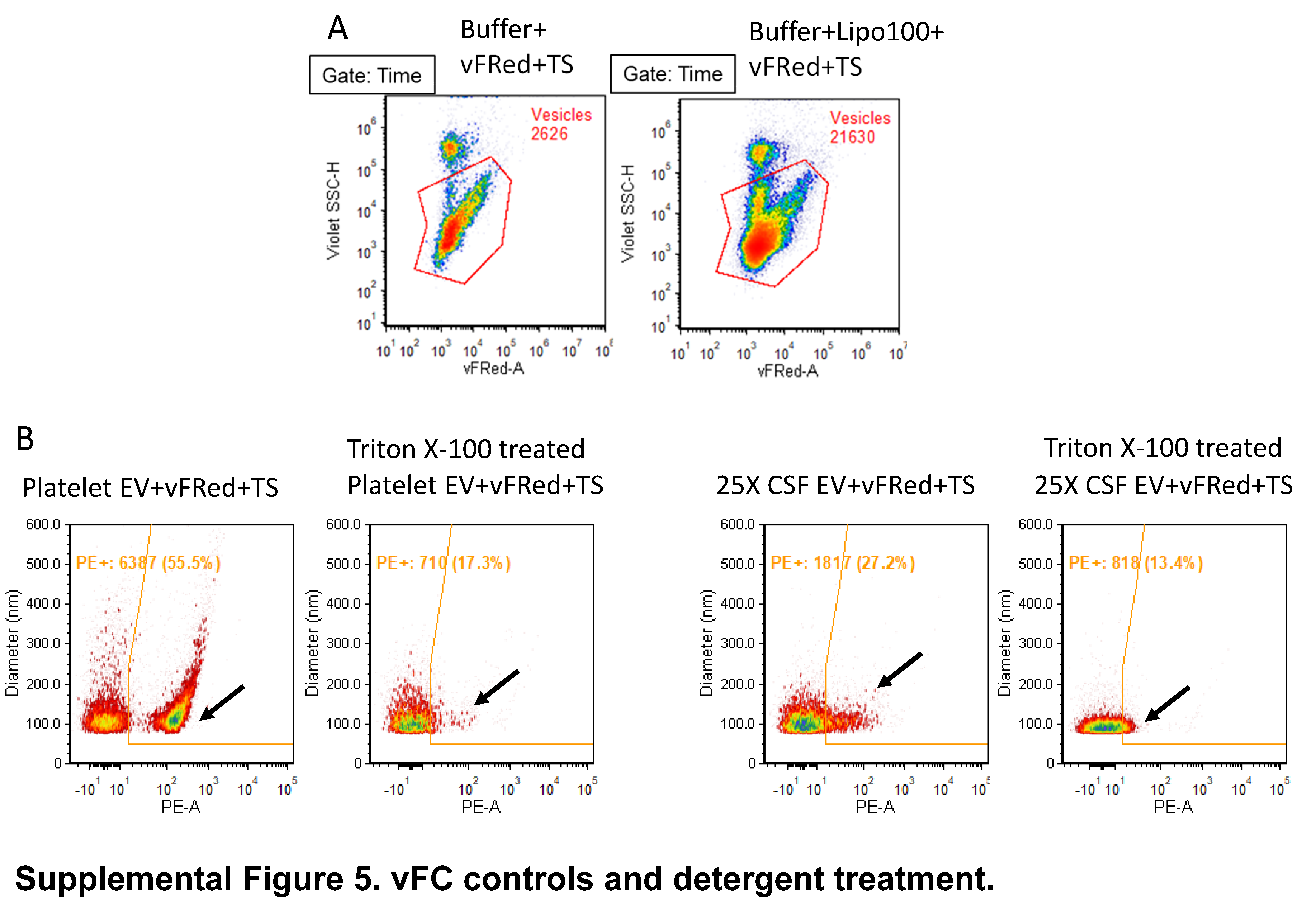

Supplement: Supplementary file 10 [file Image5.TIF]
