# Supplementary material for: Differential Effects of APOE Genotype on MicroRNA Cargo of Cerebrospinal Fluid Extracellular Vesicles in Females With Alzheimer’s Disease Compared to Males
Source: Front Cell Dev Biol. 2022 Apr 27;10:864022. doi: 10.3389/fcell.2022.864022 (PMC9092217; doi:10.3389/fcell.2022.864022)
Supplement: Supplementary file 7 [file DataSheet1.PDF]

SUPPORTING INFORMATION

Vesicle Flow Cytometry. Single vesicle flow cytometry (vFC) was performed using the vFC™ EV Analysis kit (Cellarcus Biosciences, San Diego, CA), comprised of a fluorogenic membrane stain vFRed, vesicle staining buffer, TS PE mix (CD9, CD63, CD81), Lipo100 beads, control platelet EVs, and detergent (TritonX-100). Assay measurements were performed using a CytoFlex S flow cytometer (Beckman Coulter, Pasadena, CA). Cerebrospinal fluid (CSF) samples were either assayed undiluted, or concentrated 25-fold by ultrafiltration using 100kD concentrator column (MilliporeSigma, Burlington, MA), stained with a mix of vFRed and TS PE, in a total volume of 50 µL in a 96-well v-bottom plate (82.1583.001, Sarstedt, Nümbrecht, DE) for 1 hour at room temperature, according to manufacturer's instructions. The optimal concentrations of antibody and other reagents was determined by the manufacturer via titration and provided at 10x the final staining concentration, which was between 2 and 10 nM, depending on the antibody. Stained samples were diluted 1000-fold in vesicle staining buffer (Cellarcus) and analyzed on the flow cytometer.

Flow cytometer configuration and analysis. The CytoFlex S flow cytometer used standard filters configured to measure violet side scatter (VSSC) as described in the CytoFLEX "Instructions for Use" document (https://www.beckman.com/techdocs/B49006AP/wsr-168786). Briefly, the Violet 405nm filter is placed in position 2, the Violet 450nm filter in position 3, and an unused filter in position 1. Sample was introduced at the HIGH sample flow rate (60 □L/sec, and data acquisition was triggered by the 488 nm-excited red fluorescence of the membrane stain (488-690/50 channel), with a threshold set to accept ~2 events/second with a buffer-only sample, and ~20 events/sec for a buffer +vFRed sample. Samples were analyzed for 120 seconds.

CytoFlex Instrument characterization and calibration. Prior to vFC, the CytoFlex S instrument was setup and calibrated using nanoRainbow beads (Cellarcus) that are 500 nm beads with three fluorescence intensities plus a blank using the Cellarcus "vFC Instrument Setup" document: (https://www.cellarcus.com/datasheets/vFC%20Instrument%20Setup-CytoFLEX.pdf). The results demonstrated acceptable separation in accordance with Cellarcus guidelines. The peak 4 bead population's coefficient of variation was less than 6% and the staining index (SI) was calculated to be greater than 3.0 for both the FITC and PE laser channels, (**Figure S1**). Calibration of the fluorescent channels was performed using FCS Express 7 (De Novo Software, Pasadena, CA) with the vCAL bead report layout provided by Cellarcus per the vendor instructions (**Figure S2**).

Lipo100 vesicle standard calibration. Lipo100 beads were stained, diluted, and assessed by following the Cellarcus "vFC Assay Calibration using Lipo100 Vesicle Standard" document: (https://www.cellarcus.com/datasheets/vFC%20Protocol%200.2%20-%20Lipo100%20Vesicle%20Size%20Calibration.pdf). Lipo100 data was analyzed using the FCS Express 7 (De Novo Software) vesicle layout provided by Cellarcus. The Lipo100 population estimated diameter ranged from 75nm to 300nm, with a median of 108nm and a mean of 120nm, all within the acceptable range according to Cellarcus guidelines (**Figure S3**). In addition to the Lipo100 beads, platelet EVs provided as a positive control in the vFC kit were also analyzed and reported as a mean diameter of ~150nm (**Figure S3**), consistent with known platelet EV diameter ranges and further validation of the calibration procedure.

Gating. Data were analyzed using FCS Express 7 (De Novo Software). Gating was performed via three metrics; time, fluorescence, and light scatter intensity. The first 20 seconds of data were discarded via a Time gate (**Figure S4A**) due to a consistent but unexplained background event anomaly observed on several different CytoFlex instruments. The remaining 100 seconds

of data was gated using a plot of Membrane Fluorescence-Area vs Membrane Fluorescence-Height to (**Figure S4B**) exclude certain background events that could be identified by their lower signal pulse area and widths. These events were further gated (Vesicle gate) to include events with membrane fluorescence and light scatter intensity characteristic of EVs, and to exclude high light scatter intensity background events (**Figure S4C**). Event counts in the Vesicle gate for the 100 □l of analyzed stained CSF were used to estimate the concentrations in CSF, after accounting for the pre-stain ultrafiltration concentration and post-stain dilution steps.

Controls for single vesicle analysis. The specificity of single vesicle analysis was evaluated by several control measurements. Buffer + vFRed + TS PE mix showed low levels of background events: ~2,600 in ~100 seconds of gated data compared to Buffer + Lipo100 + vFRed + TS PE mix, which had ~22,000 events in ~100 seconds (**Figure S5A**). Detergent treatment (0.05% Triton X-100) of the TS stained control platelet EVs and the 25X CSF sample, resulted in lysis of >88% and >72% of gated events, respectively (**Figure S5B**), indicating most detected events were detergent-labile as expected for EVs. Immunofluorescence negative controls included the Lipo100 vesicle standard, which bears no antigen (**Figure S6A)**. Immunofluorescence positive control included platelet EVs, which are known to express CD9 and CD63 (**Figure S6B**).

Supplemental Figure Legends

Supplemental Figure 1. Flow Cytometer Characterization. NanoRainbow beads were used to assess the CytoFlex S ability to resolve 4 distinct bead populations in the (A) vFRed and (B) PE laser channels. The brightest bead peak (M4) should have a coefficient of variation (CV) below 6%. The separation index (SI), calculated by the FCS vCal Layout provided by Cellarcus with equation ((M2-M1)/SD)) x 0.5, was used to ascertain the instrument's ability to resolve dim bead populations. As per Cellarcus guidelines, an acceptable SI should be greater than 3.0.

Supplemental Figure 2. Flow Cytometer Laser Calibration. Singlet nanoRainbow beads were gated; the 4 nanoRainbow bead populations with corresponding mean fluorescence intensity (MFI) values can be seen in the uncalibrated vFRed histogram. The log transformed MFI values were then plotted against known log transformed vFRed surface area (SA) values provided by Cellarcus (values vary from Lot to Lot but are considered nominal) to determine linear regression. The corresponding Y=mx+b slope calculation was then used to generate a calibration file (.cal) used for all subsequent vFC measurements. Calibrated histograms display the correct relationship between surface area and diameter.

Supplemental Figure 3. Assay performance using vFC kit controls and a CSF sample. (A) Lipo100 beads were used to assay instrument performance following nanoRainbow bead calibration. Typical results for Lipo100 beads have a diameter distribution between 50-300nm, with a median of 100-130nm and a mean between 120-150nm. (B) The platelet EV control sample demonstrated a typical size distribution with a mean diameter of ~150nm. (C) The 25X CSF sample demonstrated a slightly tighter distribution and somewhat smaller mean size (110nm) as compared to the platelet EV population.

Supplemental Figure 4. Vesicle Gating. (A) Time gate which excludes the first 20 seconds to eliminate initial background observed at the beginning of each assay. (B) The remaining 100 seconds is gated to exclude background events that could be identified by their lower signal pulse area and widths. (C) Vesicle gate to include events with membrane fluorescence and light scatter intensity characteristic of EVs, and to exclude high light scatter intensity background events that are often observed above the main particle populations. Gating is shown for buffer, Lipo100 beads, control platelet EVs, and for the 25X concentrated CSF sample stained with vFRed and the TS PE mix (CD9, CD63, CD81).

Supplemental Figure 5. vFC controls and detergent treatment. (A) Buffer only with the TS PE mix (CD9, CD63, CD81) resulted in far less events as compared with the Lipo100 control beads. (B) Platelet EVs and 25x concentrated CSF were stained with vFRed and the TS PE mix then treated with 0.05% TritonX-100, which resulted in greater than 88% loss of TS+ events for platelet EVs and greater than 72% in the 25X concentrated CSF sample, indicating the presence of detergent labile vesicles.

Supplemental Figure 6. vFC antibody negative and positive controls. (A) Lipo100 beads stained with vFRed and TS PE mix (CD9, CD63, CD81) served as the negative control because they lack tetraspanin antigen. Note negligible staining with the TS PE mix in the PE gate. (B) Platelet EVs known to express CD9 and CD63 were stained with vFRed and TS PE mix served as the positive staining control. Note robust TS+ population in the PE gate.
