# Supplementary material for: Differential Effects of APOE Genotype on MicroRNA Cargo of Cerebrospinal Fluid Extracellular Vesicles in Females With Alzheimer’s Disease Compared to Males
Source: Front Cell Dev Biol. 2022 Apr 27;10:864022. doi: 10.3389/fcell.2022.864022 (PMC9092217; doi:10.3389/fcell.2022.864022)
Supplement: Supplementary file 9 [file DataSheet2.docx]

**Cytometry Part A**

**Author Checklist: MIFlowCyt-Compliant Items**

| **Requirement** | **Please Include Requested Information** |
| --- | --- |
| 1.1. Purpose | The purpose of this study was to determine an approximate size, count, and percentage of tetraspanin positive vesicles in neat and concentrated total cerebral spinal fluid (CSF). |
| 1.2. Keywords | Extracellular vesicle, exosome, microvesicle, CSF, cerebral spinal fluid |
| 1.3. Experiment variables | Vesicle flow cytometry (vFC) |
| 1.4. Organization name and address | Oregon Health and Science University (OHSU)  Portland, OR 97239-3098 |
| 1.5. Primary contact name and email address | Ursula Sandau, sandauu@ohsu.edu |
| 1.6. Date or time period of experiment | May 2021 – January 2022 |
| 1.7. Conclusions | These studies indicate that CSF is suitable for characterization via vFC when pre-concentrated by a factor of at least 25-fold. Additionally, the results demonstrate at least 20-30% of detected particles observed during vFC are positive to a pool of tetraspanin markers (CD9, CD63, and CD81). |
| 1.8. Quality control measures | Instrument performance was characterized using Lipo100 beads whose intensity had been calibrated in units of MESF, multi-intensity multifluorophore beads (vCal nanoRainbow, Cellarcus). EV analysis by vesicle flow cytometry (VFC) was conducted and reported as suggested by the MIFlowCyt-EV guidelines (See attached checklist). |
| 2.1.1.1. (2.1.2.1., 2.1.3.1.) Sample description | Human CSF |
| 2.1.1.2. Biological sample source description | Remnant CSF was purchased from the biorepository, Biochemed. CSF was drawn by lumbar puncture. |
| 2.1.1.3. Biological sample source organism description | 2 adult male and 2 adult female control participants with no history of neurological defect. The ages of the donors ranged from 65-75 years old. This study utilized an equal volume pool from 1 male and 1 female to generate ~1 mL of mixed CSF sample. |
| 2.1.2.2. Environmental sample location | Details not provided by biorepository. |
| 2.3. Sample treatment description | Samples were drawn, immediately frozen and stored at –80C until the experiment was performed. |
| 2.4. Fluorescence reagent(s) description | Table S1, Supporting Information |
| 3.1. Instrument manufacturer | Beckman Coulter CytoFlexS |
| 3.2. Instrument model | CytoFlex S |
| 3.3. Instrument configuration and settings | The CytoFlex flow cytometer with stock filters (see table below) was configured to measure violet side scatter (VSSC) as described in the CytoFLEX Instructions for Use (https://www.beckman.com/techdocs/B49006AP/wsr-168786). Briefly, the Violet 405nm filter is placed in position 2, the Violet 450nm filter in position 3, and an unused filter in position 1. The gain on all scatter channels was set to 100, the gain on all fluorescence channels was set to 1000.  Detector Laser-Center/width Name  V-1 405-780/60  V-2 405-405/10 VSSC  V-3 405-450/45 BV-421  V-4 405-525/40  V-5 405-610/20  V-6 405-660/20  R-1 640-660/20  R-2 640-712/25  R-3 640-780/60  B-1 488-488/8 SSC  B-2 488-525/40 FITC  B-3 488-690/50 vFRed  Y-1 561-561/10  Y-2 561-610/20  Y-3 561-585/42 PE  Y-4 561-690/50  Y-5 561-780/60 PECy7 |
| 4.1. List-mode data files | *We recommend all authors to submit their data files to [http://flowrepository.org](http://flowrepository.org/) and to make them available for the peer-review process. If you have done so, please let us know by inserting the following codes (replace the red text):  In process |
| 4.2. Compensation description | No compensation was performed |
| 4.3. Data transformation details |  |
| 4.4.1. Gate description | Data were analyzed using FCS Express 7 (De Novo Software). The first 20 seconds of data were discarded via a Time gate (SFigure 2) due to a consistent but unexplained background event anomaly observed on several different CytoFlex instruments. The remaining 100 seconds of data, corresponding to 100 μL of measured sample, and a plot of Membrane Fluorescence-A vs Membrane Fluorescence-W used to set a gate (SFigure 2) excluding certain background events that could be identified by their lower signal pulse area and widths. These events were further gated to include events with membrane fluorescence and light scatter intensity characteristic of EVs, and to exclude high light scatter intensity background events that have been noted in certain samples (SFigure 2). |
| 4.4.2. Gate statistics | EV immunofluorescence data was analyzed to calculate the median fluorescence intensity (MFI) of the entire EV population, the number of EVs with immunofluorescence positive above gate position at the upper threshold of an unstained sample (<0.5% “positive”), and the (MFI) of these positive EVs. |
| 4.4.3. Gate boundaries | EV immunofluorescence data was analyzed to calculate the median fluorescence intensity of the entire EV population, the number of EVs with immunofluorescence positive above gate position at the upper threshold of an unstained sample (<0.5% “positive”), and the (MFI) of these positive EVs. |

**Notes**

Feel free to use more space than allocated.

You can embed graphics/figures in this document, if needed.

Please make sure to save the document in Microsoft Word version 2003 or older, before uploading to ScholarOne Manuscripts. When uploading this checklist to ScholarOne Manuscripts, please choose the “Supplementary Material for Review” category.

Please note that if your paper is accepted, the checklist will be published as an Online Supporting Information.

For any questions, please contact the Cytometry Part A editorial office at [Cytometrya@wiley.com](mailto:Cytometrya@wiley.com).
